# Linking global drivers of agricultural trade to on-the-ground impacts on biodiversity

**DOI:** 10.1073/pnas.1905618116

**Published:** 2019-10-28

**Authors:** Jonathan M. H. Green, Simon A. Croft, América P. Durán, Andrew P. Balmford, Neil D. Burgess, Steve Fick, Toby A. Gardner, Javier Godar, Clément Suavet, Malika Virah-Sawmy, Lucy E. Young, Christopher D. West

**Affiliations:** ^a^Stockholm Environment Institute York, Department of Environment and Geography, University of York, York YO10 5NG, United Kingdom;; ^b^Luc Hoffmann Institute, WWF International, 1196 Gland, Switzerland;; ^c^Conservation Science Group, Department of Zoology, University of Cambridge, Cambridge CB2 3QZ, United Kingdom;; ^d^United Nations Environment Programme World Conservation Monitoring Centre (UNEP-WCMC), Cambridge, CB3 0DL, United Kingdom;; ^e^Center for Macroecology, Evolution, and Climate, University of Copenhagen, 2100 Copenhagen, Denmark;; ^f^Stockholm Environment Institute, 115 23 Stockholm, Sweden;; ^g^Geography Department, Humboldt-Universität zu Berlin, Alfred-Rühl-Haus, 12489 Berlin, Germany;; ^h^WWF UK, Science, The Living Planet Centre, Rufford House, Woking GU21 4LL, United Kingdom

**Keywords:** supply chain, agricultural commodity, biodiversity impacts, telecoupling, species

## Abstract

Consumption of globally traded agricultural commodities like soy and palm oil is one of the primary causes of deforestation and biodiversity loss in some of the world’s most species-rich ecosystems. However, the complexity of global supply chains has confounded efforts to reduce impacts. Companies and governments with sustainability commitments struggle to understand their own sourcing patterns, while the activities of more unscrupulous actors are conveniently masked by the opacity of global trade. We combine state-of-the-art material flow, economic trade, and biodiversity impact models to produce an innovative approach for understanding the impacts of trade on biodiversity loss and the roles of remote markets and actors. We do this for the production of soy in the Brazilian Cerrado, home to more than 5% of the world´s species. Distinct sourcing patterns of consumer countries and trading companies result in substantially different impacts on endemic species. Connections between individual buyers and specific hot spots explain the disproportionate impacts of some actors on endemic species and individual threatened species, such as the particular impact of European Union consumers on the recent habitat losses for the iconic giant anteater (*Myrmecophaga tridactyla*). In making these linkages explicit, our approach enables commodity buyers and investors to target their efforts much more closely to improve the sustainability of their supply chains in their sourcing regions while also transforming our ability to monitor the impact of such commitments over time.

## Significance

Agricultural commodity production causes significant biodiversity losses, yet our globalized supply chains mean that these losses are incurred far from the places of eventual consumption. Public and private sector actors are making an increasing number of commitments to reduce their environmental impacts; to date, however, we have had limited understanding of 1) impacts at high spatial and taxonomic resolution and 2) particular consumption drivers and supply chain actors mediating trade and consumption. Without these, it is difficult to devise solutions. We link 3 state-of-the-art models to provide practical insights on the impacts of soy grown in the Brazilian Cerrado, an exceptionally biodiverse savannah that hosts some 5% of the world’s species.

Species are being lost at 1 to 2 orders of magnitude above background rates (1), with greatest losses resulting from habitat conversion and degradation, particularly appropriation for agriculture (2–4). Much of the impact of food crop production in biodiverse tropical regions is associated with commodities destined for export (5), and as much as 80 to 99% of the biodiversity impact of food crop consumption in industrialized countries is incurred abroad (5). Work linking biodiversity threats to global financial flows at the country level indicates that at least 30% of threats to globally threatened species are linked to international trade (6–8). Growing recognition of the role of global consumption in driving remote environmental damage elsewhere (9–11) has led to a number of private- and public-sector commitments to reduce these impacts, particularly in agricultural commodity supply chains (12). However, our ability to monitor in practically useful detail whether governments or businesses are making progress toward these commitments has been limited.

To devise and monitor solutions for sustainable production and consumption we need to know the location of production areas to a high degree of spatial accuracy and understand the biodiversity impacts of production in these places. Crucially, we must also understand how impacts are connected to globalized supply chains and the key actors involved (13). Progress on sustainability in supply chains will need clear and measurable targets, pathways to achieve them, and accountability (12, 14). Moreover, commitments of different stakeholders do not operate in isolation and when aligned can reinforce one another. However, the lack of methods and data to integrate policy and business perspectives prevents the design and implementation of strategies to create opportunities or regulate for more sustainable business (12, 15).

Here we combine state-of-the-art material flow, economic, and biodiversity models that link demand, trade, production, and impact. We use a species-level estimate of loss, which allows us to differentiate habitats that host the most vulnerable species from those that do not but which would appear similar or identical if broader classifications (e.g., “forest” or “natural vegetation”) were used. Our results reveal the impacts of agricultural commodity trade on biodiversity with unprecedented spatial, sectoral, operational, and taxonomic resolution.

We use our framework to answer 4 questions that together provide information for reducing biodiversity losses associated with agricultural commodity demand. First, which countries and sectors drive impacts? Understanding the role of specific consumption patterns and the responsibilities of consumers around the globe helps inform national and international policy making. Second, what are the relative roles of different commodity traders? Detailed supply chain information can help to identify and develop partnerships for solutions. Third, what are the impacts on high-profile species and important species assemblages? Highly resolved information on biodiversity impacts can galvanize support from consumer groups and provide information for particular interventions around specific species and risk hot spots. Fourth, how do government and private commitments overlap? Understanding the commitments of diverse actors along the supply chain can help identify where commitments coincide and hence where actions might be aligned to reinforce one another.

We work through our framework using the example of Brazilian soy production. Brazil is one of the world’s largest producers and exporters of soy, a globally important commodity embedded within many food products, particularly because of its use as a source of protein in animal feed. In Brazil, soy production is closely associated with the Cerrado (16, 17), which is the largest savannah region in South America and hosts some 5% of global biodiversity, including over 4,800 plant and vertebrate species found nowhere else (18). It is also one of the world’s most important frontiers of agricultural expansion, with many of its species facing dire threat (16–20). Our approach produces insights into the connections between markets, soy traders, and biodiversity losses at the point of production. We consider these in the context of 2 high-profile collective commitments: the New York Declaration on Forests, a voluntary declaration by private-, public-, and third-sector parties with a commitment to end forest loss by 2030 (21), and the Amsterdam Declaration, a commitment by 7 European countries to eliminate deforestation from agricultural commodity chains (22). These commitments are a recognition that things need to change; meeting them, however, requires a dramatic scaling up of action.

## Results

### Which Countries and Sectors Are Driving Impacts?

Information that identifies the relative roles of different countries—and sectors within them—can guide coherent action among consumer nations to drive more sustainable production practices and provision of support to key industry actors (6). The top 10 countries importing embedded soy from the Cerrado are Asian, European, and North American (Table 1). However, while international demand, especially from China, drives more than half of soy’s impacts on endemic Cerrado biodiversity, the domestic market is responsible for the greatest share of any country, with consumption across all of Brazil driving 45% of soy-related impacts (Table 1 and *SI Appendix*, Table S1). We consider these findings against country-level commitments to 2 key declarations that aim to support companies in eliminating deforestation from agricultural commodity supply chains. The first is the New York Declaration on Forests. This has been signed at the national or local government level by most of the countries with the greatest soy-linked biodiversity impacts in the Brazilian Cerrado, but the 2 countries with the greatest impact are notably absent (Table 1). The second is the Amsterdam Declaration, for which 5 of the 7 European signatories are among the top 10 importers of soy-driven biodiversity impacts in the Cerrado: Italy, France, Germany, the United Kingdom, and the Netherlands (Table 1).

**Table 1.** The countries whose embedded consumption of soy from the Cerrado in 2011 is estimated to have the greatest impact on endemic biodiversity (domestic plus top 10 international consuming countries)

Consuming region	Relative impact	Relative impact/mass consumed	Commitment
Brazil	44.9%	0.87	*
China	22.0%	0.38	
Japan	2.9%	0.52	NYDF
Germany	2.7%	0.49	NYDF/AD
Spain	2.5%	0.61	*
Thailand	2.3%	0.55	
United States	1.9%	0.36	NYDF
United Kingdom	1.8%	0.46	NYDF/AD
France	1.8%	0.33	NYDF/AD
Netherlands	1.4%	0.60	NYDF/AD
Italy	1.2%	0.87	AD

Relative impact per unit mass of soy consumed from 0 (no impact) to 1 (greatest observed impact across all consuming regions). We highlight country commitments to the New York Declaration on Forests (NYDF) and Amsterdam Declaration (AD). Asterisks indicate local, but not national, government signatories to NYDF. See also *SI Appendix*, Table S1.

**Fig. 1.**
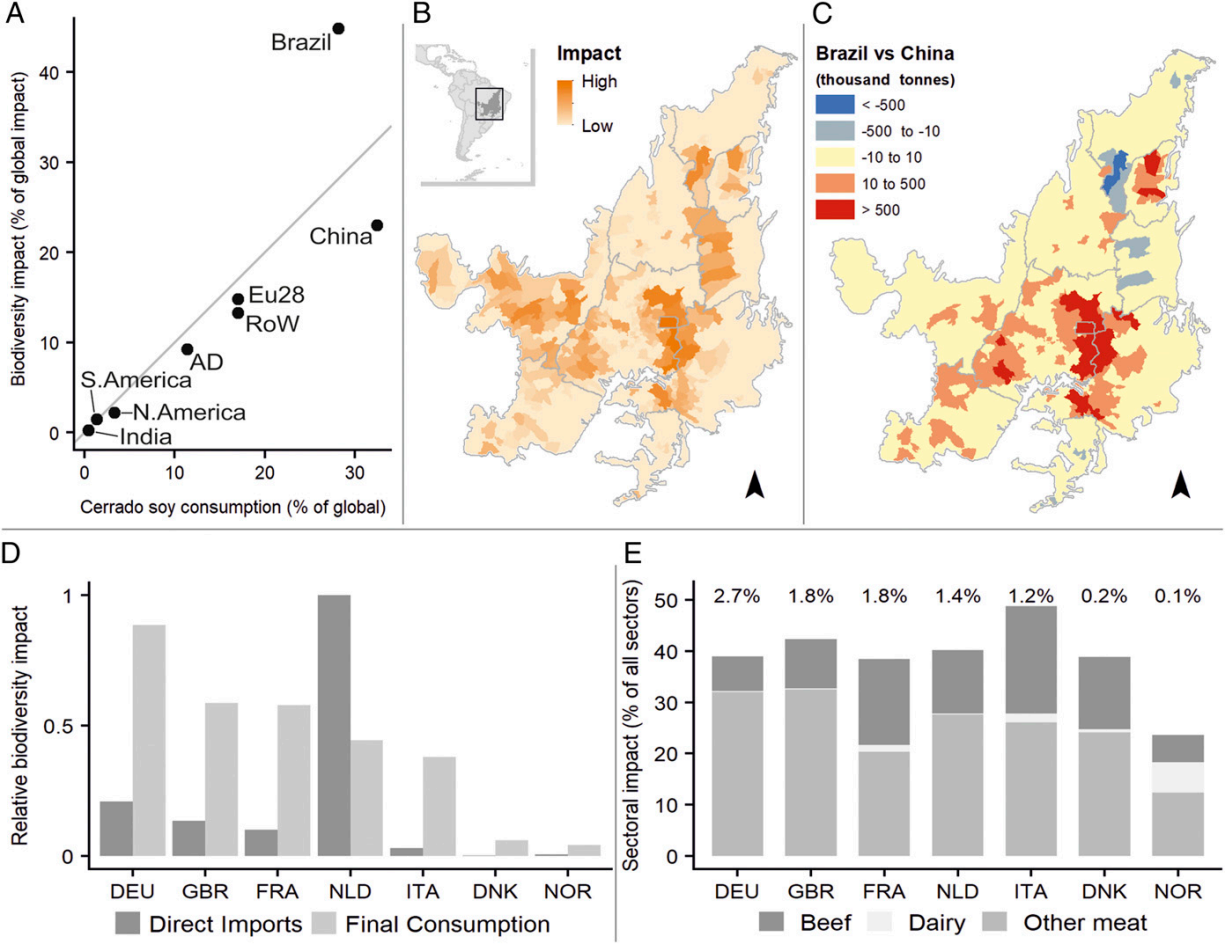
(*A*) Impact of Cerrado-sourced soy on endemic biodiversity (as a percentage of global impacts of soy in the Cerrado), plotted against embedded consumption of Cerrado-sourced soy (as a percentage of global Cerrado-sourced soy consumption) for the 7 AD countries, Brazil, the countries of the European Union (EU28), China (including Hong Kong and Taiwan), India, North America, South America, and the rest of the world (RoW). Gray line indicates mean global impact per unit of soy consumption. (*B*) Spatial pattern of our endemic biodiversity loss index within the Cerrado during the period 2000 to 2010. (*C*) Difference (tons) between production for domestic consumption (all Brazil) and Chinese consumption. Negative values (blue) are municipalities where production for Chinese consumption exceeds production for Brazil. Positive values (orange/red) are municipalities where production for Brazilian consumption exceeds production for China. (*D*) Comparison of the relative soy-attributed biodiversity impact that is directly imported to AD countries and impact that is attributed to final consumption within those countries (i.e., the latter accounts for both reexports and embedded consumption). (*E*) Sectoral and countrywise differences for AD countries showing the relative impact of 3 key soy-linked sectors as a percentage of each country’s consumption of soy across all sectors combined. The value above the bar indicates the relative importance of each country to global biodiversity impacts of Cerrado-sourced soy.

Alongside the amount of soy consumed, the impact per unit consumed also varies greatly between countries. Brazil and Italy, for example, have over twice the impact per unit of soy consumed than China, France or the United states. The 2 largest consuming countries, Brazil and China, consume similar amounts of soy from the Cerrado but show particularly high and low impacts per ton, respectively (Fig. 1*A*). These differences arise from differences in biodiversity losses in the municipalities from which particular supply chains source soy. By combining high-resolution trade data with impacts on biodiversity we find that Brazilian consumer demand was met to a greater extent by municipalities in the central and southern Cerrado, where endemic richness is higher and impacts are thus greater (Fig. 1 *B* and *C* and *SI Appendix*, Fig. S1). Chinese demand, on the other hand, was met from a more tightly concentrated area in the northeast (Fig. 1*C*).

By linking direct material flows to global financial data, our approach also captures both the reexports of soy (for example, much of the soy consumed in Europe arrives via ports in the Netherlands, from where it is reexported) and the consumption of soy embedded in other products, such as in meat fed on soy-derived feed. The Netherlands is a globally important trade hub, receiving much of the soy coming directly from Brazil into the European Union (EU) (Fig. 1*D*). However, tracking supply chains only to the country of first import greatly overestimates the country’s role as a driver of biodiversity loss, while for other Amsterdam Declaration (AD) countries their role is substantially underestimated unless we consider reexports and embedded consumption of soy (Fig. 1*D*).

Sectoral drivers of biodiversity loss vary markedly between countries. In the case of AD countries, particularly Germany and the United Kingdom, our results highlight the importance of “other meat” (primarily pig and poultry) consumption (Fig. 1*E*). For Italy and Norway, on the other hand, dairy and beef sectors contribute a relatively larger proportion of their biodiversity footprint.

### What Are the Relative Roles of Different Traders?

For the Cerrado we estimate that between 2000 and 2010, 33% of soy’s impacts on endemic species were in Goiás State, which occupies just 16% of the biome (*SI Appendix*, Fig. S2 and Table S2). Of 41 traders exporting soy from Goiás in 2011, the top 10 account for 91% of exports. Disaggregating the data to the municipality level reveals the highly clustered nature of company operations (*SI Appendix*, Fig. S2). The largest exporter in each municipality accounts for a mean of 97% of exports. Just 5 traders account for all soy exports from the 3 most heavily affected municipalities, which together incur 56% of the state’s soy-driven biodiversity losses but cover <4% of the area.

### What Are the Impacts on High-Profile Species and Important Species Assemblages?

**Fig. 2.**
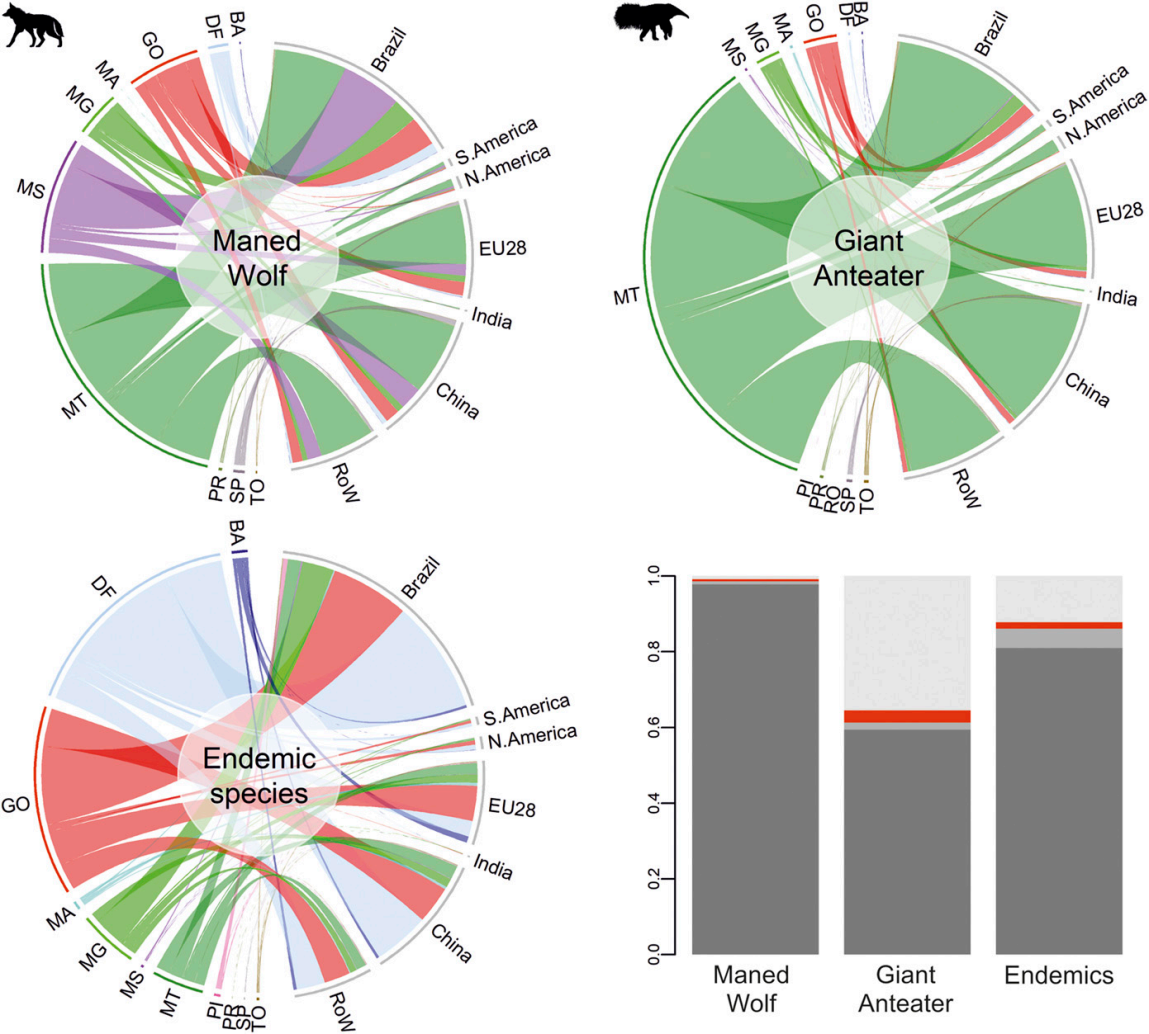
Chord diagrams showing impacts on likelihood of persistence due to soy expansion between 2000 and 2010 for 2 charismatic species (*Top*) and for all endemics (*Bottom Left*). Losses are calculated for each municipality according to the total embedded flows of soy and then aggregated to state level for visualization. Chords show the flow from states on the left-hand side (BA = Bahia, dark blue; DF = Distrito Federal, gray; GO = Goiás, red; MA = Maranhão, cyan; MG = Minas Gerais, light green; MS = Mato Grosso do Sul, purple; MT = Mato Grosso, dark green; PI = Piauí, pink; PR = Paraná, dark olive green; RO = Rondônia, brown; SP = São Paulo, dark gray; TO = Tocantins, gold) through to the country or region of final consumption on the right-hand side (Brazil, South America, North America, European Union, India, China, and the rest of world). The proportion of remaining suitable habitat within the Cerrado for the 2 species (*Bottom Right*) and the mean for all endemic species. Light gray: suitable habitat lost from the preindustrial era to the year 2000; red: losses during the 2000 to 2010 study period (as represented in the chord diagrams); medium gray: losses between 2010 and 2014; dark gray: remaining suitable habitat in 2014.

Quantifying how consumption drives losses of charismatic, culturally important, or valuable species and habitats can raise the profile of environmental issues and bring into focus the tangible impacts and risks of sourcing from a particular area (23). The spatial and taxonomic resolution of the component models in our framework enables fine-scale, species-specific information that is typically masked in national-level analyses. To illustrate this, we compare impacts of soy-driven habitat loss on 2 iconic species, the maned wolf (*Chrysocyon brachyurus*) and giant anteater (*Myrmecophaga tridactyla*), with impacts on endemic species, and characterize these as flows from the state in which the losses occur through to the country of final consumption of the impact-linked soy (Fig. 2). This reveals some striking patterns resulting from differences between the threats facing different species and from differences in sourcing between consuming countries. For example, the majority of the EU’s impact on the maned wolf is in Mato Grosso, while for Brazil it is in other states. This has implications for the targeting of conservation interventions by downstream actors wanting to mitigate specific impacts associated with their activities. We also found that the giant anteater’s range has been more heavily impacted by past habitat loss than that of the maned wolf [which better tolerates pasture and arable land (17)] and that the EU has played a large role in recent losses, with impacts mostly arising in Mato Grosso. Unlike for the maned wolf and giant anteater, losses in Goiás and Distrito Federal dominate impacts across endemic species, largely due to the high number of endemics, particularly plants, found in these states (Fig. 2 and *SI Appendix*, Fig. S1).

### How Do Government and Private Commitments Overlap?

**Fig. 3.**
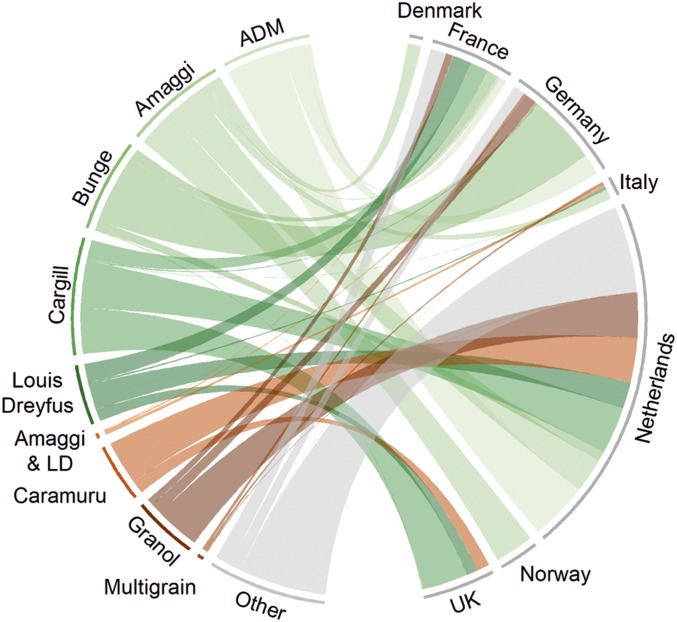
Alignment of government commitments with sustainability goals of key traders. Chord diagram representing direct soy trade from the Brazilian Cerrado to the 7 countries of the Amsterdam Declaration from the largest traders in 2011 (companies shown were among the top 3 traders in 2011 for at least one of the countries; companies trading smaller volumes are aggregated and shaded gray). Green shaded chords indicate exports via companies with zero-deforestation commitments; orange and brown shades indicate no such commitment (data from company websites as of December 2018).

In 2011, companies with zero-deforestation commitments were responsible for ∼80% of soy imports for France, Germany, and the United Kingdom (Fig. 3 and *SI Appendix*, Table S3). The Netherlands, on the other hand, has a more diverse supplier base, with ∼50% supplied by traders with zero-deforestation commitments.

## Discussion

It is encouraging that many of the countries and traders most exposed to the risks of deforestation and biodiversity loss in their supply chains have joined high-profile declarations to eliminate deforestation from their supply chains (e.g., refs. 21, 22, and 24). However, company commitments to reducing deforestation in supply chains vary widely in their detail, ambition, and meaning (12, 15). Understanding alignment between government and trader commitments will help identify where action should be focused, reveal potential leverage points, and help foster coordinated solutions for international supply chains that span multiple stakeholders across the private–public interface (12, 15). If supporting companies make good on their commitments, this would in turn help governments make significant progress toward their own commitments to eliminate deforestation and may push the sustainability bar higher for smaller or newer actors in the European market. Within our analyses, the 2 countries with the greatest overall impacts, Brazil and China, have not yet signed key declarations at the national level (although note that Mato Grosso, an important soy-producing state within the Cerrado, has committed to its Produce, Conserve, and Include Strategy, which aims to reduce Cerrado deforestation by 95% and to restore habitat; ref. 25).

Attributing impacts to the country of first import can both severely underestimate (e.g., Denmark and Norway) and overestimate (e.g., the Netherlands) impacts attributed to a country’s final consumption. However, in the same way that identifying key traders operating within the supply chain can help identify important opportunities for intervention, so too can identifying the most significant hubs for trade. The Netherlands is the largest importer of soy in Europe and the second-largest exporter of agricultural products in the world (26). It also processes ∼25% of its soy imports to produce animal feed (26). These factors underlie its central role in the global soy value chain and its founding role in the Amsterdam Declaration. The Netherlands could continue to exert disproportionate influence on trading companies and buyers as a convening power and focal point of private–public dialogue and partnerships (e.g., Dutch Soy Coalition, Dutch Soy Working Group, and the Dutch Soy Platform Initiative) (24, 26). The Dutch government has also provided support to processors and buyers that invest in certification (Soy Fast Track Fund), as well as to farmers to enable them to produce more sustainable soy (Farmer Support Program) (26). In addition, governments have an important convening and financing role to play in establishing sustainable finance, including provision of credit lines to farmers who adhere to higher sustainability criteria or support to scale up innovative solutions to sustainability challenges (e.g., refs. 27 and 28). Our estimates of the impacts of final consumption highlight the substantial responsibilities too of other EU countries, such as Spain, which is not currently a signatory to the declaration but could be a focal point for targeted political influence by existing signatories (Table 1).

While the Netherlands may hold some influence because of its large trade volumes, its diverse portfolio of traders could make policy processes more complex and contested. In contrast to other AD countries a large proportion of soy exported to (and through) the Netherlands is from traders without zero-deforestation commitments. Hence, even if those with existing commitments delivered on them, this would capture just half of the Cerrado soy traded through the Netherlands (Fig. 3 and *SI Appendix*, Table S3). Working with countries that directly import substantially smaller volumes, such as the United Kingdom, France, and Germany, may help the Netherlands government to encourage currently uncommitted yet major traders such as Caramuru and Granol to sign up to targets to eliminate deforestation from their supply chains.

There are several sources of uncertainty within the models presented, for example, in modeling land cover, estimating biodiversity loss, modeling trade, and year-to-year variability of supply chains. The Trase Spatially Explicit Information on Production to Consumption Systems (SEI-PCS) model of subnational production and export is built from key government statistics and data that are compiled to calculate agricultural productivity and to collect tax revenues (29). This allows considerable confidence in this aspect of the modeling. The Input-Output Trade Analysis (IOTA) model employed in the analysis is one of several multiregional input–output models (MRIOs) that are available globally, all of which will provide somewhat different quantitative results due to differences in their construction (30). Our results are illustrative of the impacts that different countries might have, highlighting the heterogeneity that is expected across the trade system. Use of such information in risk assessment or supply chain decision making should consider the assumptions made and associated limitations of the modeling approaches. More targeted analysis (e.g., of particular supply chains looking at specific priority species) would benefit from further sensitivity analyses to explore how changes in assumptions might affect conclusions. We use 2011 trade data in our analyses that provide a snapshot of a dynamic system, particularly in the most active frontiers of agricultural expansion. Any intervention should be based on multitemporal analyses of spatial patterns and trends, as well as iterative engagement with stakeholders to ensure their accuracy and relevance. However, because of the investments in infrastructure (such as silos and crushing facilities) and knowledge and interdependencies between actors, we expect traders to stay relatively connected to particular production locations over a 3 to 5 y span, with more significant changes occurring over longer periods (refs. 20, 31, and 32; see supplementary analyses in *SI Appendix*, Figs. S3 and S4). Understanding how the data available within our framework might be used to help determine accountability for impacts occurring across a dynamic trading landscape, where impacts can occur several years prior to trading activities, deserves additional research focus.

## Conclusion

Currently, many sustainability commitments are little more than statements of intent and a recognition that things need to change (12, 15). Meeting these commitments requires collective action to be scaled up through multistakeholder partnerships, landscape-scale approaches, and public–private initiatives (12). Identifying links between the intensification and expansion of agricultural commodity production and the demand that drives it is a vital first step to engage the political and private actors with the greatest responsibility and influence. We provide a highly flexible framework for delivering a range of practical insights to stakeholders in international commodity supply chains. Businesses can use this information to understand risks in their supply chains, while civil society, consumers, and shareholders can use it to hold governments and businesses to account on their commitments. Investors too are increasingly interested in understanding investment-linked environmental and social risks (33), and this will likely increase as transparency initiatives more precisely link the environmental damage caused by commodity production to hitherto opaque financial systems underpinning it (34).

The high spatial resolution of our trade model tracking production and subnational flows is a major advance for 2 reasons: First, in enhancing the credibility and spatial representation of estimates of environmental impact and, second, in transforming our ability to devise and implement responses. For example, campaigners can use impacts on flagship species to galvanize support from consumer groups and to promote responsible consumption across supply chain actors. Higher-resolution models allow us to develop land use management strategies to target particular areas for improving yields, setting aside areas for protection in expansion landscapes, or expanding production into degraded land according to the level of endemicity or of historical impacts on biodiversity. More generally, the spatial resolution demonstrated here allows the development of more credible estimates for a suite of indicators of environmental and social impacts. This species-level metric complements, rather than replaces, other measures of biodiversity loss based on the loss of ecosystems (such as the loss of the Cerrado or deforestation) (e.g., refs. 35 and 36). Taken together, these provide a more complete picture of how the trade in a commodity such as soy drives both immediate and longer-term losses and has impacts at scales from the very local to global. It also allows assessment of complementarity or trade-offs between, for example, protecting forests versus endemic species.

Our approach is applicable to a wide range of globally traded agricultural commodities. However, to “catalyze a race to the top” (14), actors must also be supported by mechanisms that allow and recognize iterative improvements. Without such mechanisms, shedding light on sustainability problems within particular supply chains may cause actors to shift to different production regions, rather than improving practices in vulnerable areas, or to start supplying consuming regions without commitments to eliminate deforestation or where consumer pressure is currently lower (12, 15). Anticipating such “leakage” between areas, countries, and, indeed, different commodity crops is vital. In this context our ability to document country–trader relationships is likely to play an important role. Many of the biggest traders source from multiple producer countries, sell their goods globally, and have activities that span several commodities (37). This global reach may allow successful sustainability initiatives to quickly scale up to other regions and commodities. By enabling monitoring of shifts of traders between markets our framework can also help minimize leakage by ensuring that sustainability commitments apply across companies’ operations. Moreover, because of the dominant role that a relatively few traders hold as a nexus of global commodity flows (38, 39), pressure from major economies, such as the AD countries, to improve environmental standards could drive improvements to the sustainability of supply chains to other consuming regions.

## Methods

We compiled and integrated existing data sources, linking complementary approaches to derive information on consumption patterns driving species declines and shedding light on the supply chains involved (*SI Appendix*, Fig. S5). Existing MRIOs use data on intersectoral financial transactions to represent full global trade and consumption but sacrifice commodity-specific detail and spatial resolution. Conversely, material flow analyses—descriptions of the physical movement of commodities—can be used to track production and trade of individual commodities but generally capture only a portion of the supply chain (40). We therefore developed a hybridized MRIO for soy trade that combines traditional input–output analyses with highly detailed subnational material flow data from the SEI-PCS model underpinning the Trase platform (36, 41) (*SI Appendix*). We used these to tease out the activities of producers, traders, and consumers. We linked the models to estimates of species-by-species losses of suitable habitat to derive a measure of biodiversity impact that accounts for species-specific differences in range sizes, sensitivities to land use change, and historical habitat loss (17) (*SI Appendix*, Fig. S5). We focused on the impacts of soy production in 2000 to 2010 using habitat loss data for 2000 to 2010 and soy trade data for 2011. We chose this allocation period (i.e., attributing 2000 to 2010 losses to 2011) because it can take several years from initial clearing of land to eventual harvesting and selling soy.

## Supplementary Material

Supplementary File

Supplementary File
