# Supplementary material for: Linking global drivers of agricultural trade to on-the-ground impacts on biodiversity
Source: Proc Natl Acad Sci U S A. 2019 Oct 28;116(46):23202–8. doi: 10.1073/pnas.1905618116 (PMC6859333; doi:10.1073/pnas.1905618116)
Supplement: Supplementary File [file pnas.1905618116.sapp.pdf]

Supporting Information: Linking global drivers of agricultural trade to on-the-ground impacts on biodiversity

Authors: Jonathan M.H. Green[a,b], Simon A. Croft[a], América P. Durán[b,c,d], Andrew P. Balmford[c], Neil D. Burgess[d,e], Steve Fick[f], Toby A. Gardner[f], Javier Godar[f], Clément Suavet[f], Malika Virah-Sawmy[b,g], Lucy E. Young[h], Christopher D. West[a]

Institutions:

[a]Stockholm Environment Institute – York Centre, Department of Environment and Geography, University of York, YO10 5NG, United Kingdom

[b]Luc Hoffmann Institute, WWF International, Rue Mauverney 28, 1196 Gland, Switzerland.

[c]Conservation Science Group, Department of Zoology, University of Cambridge, Cambridge, CB2 3QZ, United Kingdom

[d]UN Environment Programme World Conservation Monitoring Centre (UNEP-WCMC), 219 Huntington Road, CB3 0DL, Cambridge, United Kingdom

[e]Center for Macroecology, Evolution and Climate, The Natural History Museum, University of Copenhagen, Copenhagen, Denmark

[f]Stockholm Environment Institute, Linnégatan 87D, 115 23 Stockholm, Sweden

[g]Geography Department, Humboldt-Universität zu Berlin, Alfred-Rühl-Haus, Rudower Chaussee 16, 12489 Berlin, Germany

[h]WWF UK, The Living Planet Centre, Rufford House, Brewery Road, Woking GU21 4LL, United Kingdom

Contents

Appendix 1

- Table S1. The impact of different consuming regions on endemic species (page 2)
- Figure S1. Richness of endemic and near endemic species used in this study (page 6)
- Figure S2. Trader dominance in Goiás (page 7)
- Table S2. Traders exporting from the state of Goiás (page 8)
- Table S3. Top traders exporting to Amsterdam Declaration countries (page 9)
- Table S4. List of species included in analyses and their extent of suitable habitat (page 10)
- Figure S3.
- Figure S4.

Appendix 2

- Supplementary analyses to compare 2011 snapshot with 2006-2010 average (page 20)

Appendix 3

- Supplementary methods (page 24)
    1. Study methods
    2. Trase SEI-PCS model
    3. IOTA model
    4. Re-export algorithm
    5. Biodiversity impact extension

Appendix 4

- Supplementary sensitivity analyses with results from alternative biodiversity method (page 37)

www.pnas.org/cgi/doi/10.1073/pnas.1905618116

Appendix 1

Table S1. *The impact of different consuming regions on endemic species due to embedded consumption of soy from the Cerrado. Regions are ranked from greatest to least impact and specified according to GTAP 9* (1).

	Consuming region	*GTAP code*	*Region classification*	*Commitments*	**Endemic species impact (% global)**	**Relative impact/mass consumed**	**Population (2010)**	*Per capita impact rank*	*Embedded soy (tonnes)*	*Soy consumption (% global total)*
**1**	Brazil	BRA	South America	[NYDF]	44.9	0.87	1.97E+08	1	15232107	28.2
**2**	China	CHN	East Asia		22.0	0.38	1.34E+09	38	17071040	31.6
**3**	Japan	JPN	East Asia	NYDF	2.9	0.52	1.28E+08	27	1601338	3.0
**4**	Germany	DEU	Europe - EU	NYDF + ADF	2.7	0.49	81776930	12	1646251	3.0
**5**	Spain	ESP	Europe - EU	[NYDF]	2.5	0.61	46576897	5	1183742	2.2
**6**	Thailand	THA	Southeast Asia		2.3	0.55	67208808	10	1241091	2.3
**7**	United States of America	USA	North America	NYDF	1.9	0.36	3.09E+08	63	1552402	2.9
**8**	United Kingdom	GBR	Europe - EU	NYDF + ADF	1.8	0.46	62766365	19	1155280	2.1
**9**	France	FRA	Europe - EU	NYDF + ADF	1.8	0.33	65027512	20	1587406	2.9
**10**	Netherlands	NLD	Europe - EU	NYDF + ADF	1.4	0.60	16615394	2	672552.4	1.2
**11**	Italy	ITA	Europe - EU	ADF	1.2	0.43	59277417	30	800576.3	1.5
**12**	Russian Federation	RUS	Europe		1.1	0.62	1.43E+08	56	517508.2	1.0
**13**	Saudi Arabia	SAU	Western Asia		0.9	0.65	27425676	15	396506.6	0.7
**14**	Venezuela	VEN	South America		0.7	0.97	29028033	24	222243.7	0.4
**15**	Korea Republic of	KOR	East Asia	NYDF	0.6	0.19	49554112	49	862655.8	1.6
**16**	Taiwan	TWN	East Asia		0.6	0.67	23102406	26	247897.2	0.5
**17**	Viet Nam	VNM	Southeast Asia	NYDF	0.5	0.30	86932500	64	532788.7	1.0
**18**	Iran Islamic Republic of	IRN	Western Asia		0.5	0.25	74567511	58	627203.4	1.2
**19**	Belgium	BEL	Europe - EU	NYDF	0.5	0.45	10895586	6	337036.9	0.6
**20**	Poland	POL	Europe - EU		0.5	0.60	38042794	45	226876.3	0.4
**21**	Hong Kong	HKG	East Asia		0.5	0.52	7024200	4	254743.4	0.5
**22**	Turkey	TUR	Western Asia		0.4	0.47	72326914	70	239326.5	0.4
**23**	Portugal	PRT	Europe - EU		0.3	0.44	10573100	14	229863.4	0.4
**24**	Rest of Western Asia	XWS	Western Asia		0.3	0.78	83536557	75	115560.9	0.2
**25**	United Arab Emirates	ARE	Western Asia		0.3	0.58	8270684	9	143089.5	0.3
**26**	Romania	ROU	Europe - EU		0.3	0.42	20246871	44	194232.1	0.4
**27**	Austria	AUT	Europe - EU		0.3	0.47	8363404	17	165423	0.3
**28**	India	IND	South Asia		0.3	0.32	1.23E+09	130	232572.7	0.4
**29**	Egypt	EGY	North Africa		0.2	0.30	84107606	81	243468	0.5
**30**	South Africa	ZAF	South African Customs Union		0.2	0.54	50979432	73	124145.3	0.2
**31**	Argentina	ARG	South America		0.2	0.68	41223889	68	97461.6	0.2
**32**	Czech Republic	CZE	Europe - EU		0.2	0.53	10474410	28	124577.9	0.2
**33**	Switzerland	CHE	Europe - EFTA		0.2	0.51	7824909	21	124426	0.2

	Consuming region	GTAP code	Region classification	Commitments	Endemic species impact (% global)	Relative impact/mass consumed	Population (2010)	Per capita impact rank	Embedded soy (tonnes)	Soy consumption (% global total)
**34**	Rest of North Africa	XNF	North Africa		0.2	0.27	42767051	71	221352.3	0.4
**35**	South Central Africa	XAC	South Central Africa		0.2	0.74	87892394	91	77020.35	0.1
**36**	Sweden	SWE	Europe - EU		0.2	0.36	9378126	29	156158.6	0.3
**37**	Denmark	DNK	Europe - EU	NYDF + ADF	0.2	0.44	5547683	11	125417.3	0.2
**38**	Canada	CAN	North America	NYDF	0.2	0.38	34005274	67	145559.4	0.3
**39**	Indonesia	IDN	Southeast Asia	NYDF	0.2	0.15	2.43E+08	114	332857.1	0.6
**40**	Australia	AUS	Oceania		0.2	0.34	22031750	55	148570.5	0.3
**41**	Greece	GRC	Europe - EU		0.2	0.54	11121341	42	91705.84	0.2
**42**	Chile	CHL	South America	NYDF	0.2	0.84	16993354	50	59272.1	0.1
**43**	Singapore	SGP	Southeast Asia		0.1	0.69	5076732	18	63524.61	0.1
**44**	Hungary	HUN	Europe - EU		0.1	0.45	10000023	43	90579.67	0.2
**45**	Norway	NOR	Europe - EFTA	NYDF + ADF	0.1	0.23	4889252	23	165118.4	0.3
**46**	Kuwait	KWT	Western Asia		0.1	0.92	2998083	8	40423.79	0.1
**47**	Malaysia	MYS	Southeast Asia		0.1	0.38	28112289	74	95915.75	0.2
**48**	Caribbean	XCB	Caribbean		0.1	0.16	22938512	69	225645.9	0.4
**49**	Ukraine	UKR	Europe		0.1	0.61	45870700	84	58167.88	0.1
**50**	Ireland	IRL	Europe - EU		0.1	0.47	4560155	22	74552.91	0.1
**51**	Mexico	MEX	North America	NYDF	0.1	0.38	1.17E+08	109	81190.55	0.2
**52**	Colombia	COL	South America	NYDF	0.1	0.22	45918097	93	131594	0.2
**53**	Finland	FIN	Europe - EU		0.1	0.41	5363352	33	65498.84	0.1
**54**	Israel	ISR	Western Asia		0.1	0.26	7623600	47	105229.1	0.2
**55**	Philippines	PHL	Southeast Asia	NYDF	0.1	0.44	93726624	108	56723.58	0.1
**56**	Slovakia	SVK	Europe - EU		0.1	0.64	5391428	40	39389.26	0.1
**57**	Peru	PER	South America	NYDF	0.1	0.24	29373646	83	95348.26	0.2
**58**	Nigeria	NGA	Western Africa	[NYDF]	0.1	0.22	1.59E+08	124	98419.87	0.2
**59**	Croatia	HRV	Europe - EU		0.1	0.30	4417781	41	68254.48	0.1
**60**	Bangladesh	BGD	South Asia		0.1	0.17	1.52E+08	128	106141.6	0.2
**61**	Paraguay	PRY	South America		0.1	0.66	6209877	51	26702.18	0.0
**62**	Qatar	QAT	Western Asia		0.1	0.67	1779676	13	25643.57	0.0
**63**	Jordan	JOR	Western Asia		0.1	0.62	7182390	54	26968.91	0.0
**64**	Lithuania	LTU	Europe - EU	NYDF	0.1	0.48	3097282	32	32639.38	0.1
**65**	Bulgaria	BGR	Europe - EU		0.1	0.48	7395599	59	31194.6	0.1
**66**	Oman	OMN	Western Asia		0.0	0.80	3041460	39	17761.86	0.0
**67**	Ghana	GHA	Western Africa		0.0	0.58	24512104	95	23419.41	0.0
**68**	Kazakhstan	KAZ	Western Asia		0.0	0.36	16321581	85	35244.59	0.1
**69**	Rest of East Asia	XEA	East Asia		0.0	0.31	25128568	99	40517.99	0.1
**70**	Panama	PAN	Central America	NYDF	0.0	0.36	3643222	48	34823.41	0.1
**71**	Morocco	MAR	North Africa		0.0	0.29	32409639	104	43105.05	0.1
**72**	Rest of Europe	XER	Europe		0.0	0.52	14178526	82	23301.25	0.0
**73**	Uruguay	URY	South America		0.0	0.50	3374415	46	24149.33	0.0
**74**	Central Africa	XCF	Central Africa		0.0	0.80	23488510	98	15097.74	0.0
**75**	Bahrain	BHR	Western Asia		0.0	0.58	1240862	16	19934.53	0.0
**76**	Ecuador	ECU	South America	NYDF	0.0	0.27	14934690	86	40154.33	0.1
**77**	Rest of Western Africa	XWF	Western Africa		0.0	0.51	49271641	112	20857.11	0.0

	Consuming region	GTAP code	Region classification	Commitments	Endemic species impact (% global)	Relative impact/mass consumed	Population (2010)	Per capita impact rank	Embedded soy (tonnes)	Soy consumption (% global total)
**78**	Latvia	LVA	Europe - EU		0.0	0.54	2097555	34	19674.86	0.0
**79**	Slovenia	SVN	Europe - EU	NYDF	0.0	0.54	2048583	35	19024.9	0.0
**80**	Georgia	GEO	Western Asia		0.0	0.21	3926000	52	48300.62	0.1
**81**	Luxembourg	LUX	Europe - EU		0.0	0.49	506953	3	19875.99	0.0
**82**	Pakistan	PAK	South Asia		0.0	0.25	1.71E+08	131	37955.41	0.1
**83**	Lao People's Democratic Republic	LAO	Southeast Asia		0.0	0.88	6246274	72	9632.175	0.0
**84**	Benin	BEN	Western Africa		0.0	0.81	9199259	79	10165.89	0.0
**85**	Rest of Eastern Africa	XEC	Eastern Africa		0.0	0.44	61436282	125	18603.78	0.0
**86**	Cyprus	CYP	Europe - EU		0.0	0.56	1112607	25	14581.79	0.0
**87**	New Zealand	NZL	Oceania		0.0	0.34	4350700	62	23432.66	0.0
**88**	Rest of Southeast Asia	XSE	Southeast Asia		0.0	0.41	51265487	122	18997.46	0.0
**89**	Tunisia	TUN	North Africa		0.0	0.32	10639931	87	23684.37	0.0
**90**	Bolivia	BOL	South America		0.0	0.62	9918242	88	11246.66	0.0
**91**	Rest of Former Soviet Union	XSU	Western Asia		0.0	0.40	41291240	119	16994.85	0.0
**92**	Dominican Republic	DOM	Caribbean	NYDF	0.0	0.41	9897985	89	16242.09	0.0
**93**	Mozambique	MOZ	Eastern Africa		0.0	0.34	24221405	107	19599.48	0.0
**94**	Estonia	EST	Europe - EU	NYDF	0.0	0.42	1331475	37	15544.18	0.0
**95**	Belarus	BLR	Europe		0.0	0.51	9490583	90	12118.25	0.0
**96**	Cambodia	KHM	Southeast Asia		0.0	0.40	14308740	103	15305.86	0.0
**97**	Zimbabwe	ZWE	Eastern Africa		0.0	0.45	14086317	102	13406.38	0.0
**98**	Azerbaijan	AZE	Western Asia		0.0	0.72	9054332	92	7905.794	0.0
**99**	Albania	ALB	Europe		0.0	1.00	2913021	60	5699.41	0.0
**100**	Malta	MLT	Europe - EU		0.0	0.44	414508	7	12277.28	0.0
**101**	Kyrgyzstan	KGZ	Western Asia		0.0	0.38	5447900	78	13390.95	0.0
**102**	Rest of Oceania	XOC	Oceania		0.0	0.38	10144450	101	11724.64	0.0
**103**	Rest of South Asia	XSA	South Asia		0.0	0.44	29897808	126	8458.607	0.0
**104**	Puerto Rico	PRI	Caribbean		0.0	0.36	3721525	77	10338.67	0.0
**105**	Kenya	KEN	Eastern Africa	NYDF	0.0	0.36	41350152	129	9655.273	0.0
**106**	Cameroon	CMR	Western Africa		0.0	0.37	19970495	120	8652.446	0.0
**107**	Senegal	SEN	Western Africa		0.0	0.41	12916229	110	7969.962	0.0
**108**	Rest of South America	XSM	South America		0.0	0.56	1509692	57	5681.534	0.0
**109**	Armenia	ARM	Western Asia		0.0	0.99	2877311	76	3122.801	0.0
**110**	Sri Lanka	LKA	South Asia		0.0	0.35	20119000	121	8867.441	0.0
**111**	Cote d'Ivoire	CIV	Western Africa	NYDF	0.0	0.53	20401331	123	5749.688	0.0
**112**	Trinidad and Tobago	TTO	Caribbean		0.0	0.54	1328100	61	4692.711	0.0
**113**	Jamaica	JAM	Caribbean		0.0	0.73	2817210	80	3418.083	0.0
**114**	Costa Rica	CRI	Central America	NYDF	0.0	0.43	4545280	96	5834.416	0.0
**115**	Guatemala	GTM	Central America		0.0	0.40	14630417	117	6220.229	0.0
**116**	Togo	TGO	Western Africa	NYDF	0.0	0.43	6502952	106	5144.282	0.0
**117**	Mauritius	MUS	Eastern Africa		0.0	0.29	1250400	65	7631.541	0.0
**118**	Tanzania United Republic of	TZA	Eastern Africa		0.0	0.35	46098591	135	5915.478	0.0
**119**	Guinea	GIN	Western Africa		0.0	0.43	10794170	118	4279.207	0.0
**120**	Rest of EFTA	XEF	Europe - EFTA		0.0	0.41	354044	36	4293.216	0.0
**121**	Rest of Eastern Europe	XEE	Europe		0.0	0.60	3562045	100	2842.019	0.0

Consuming region	GTAP code	Region classification	Commitments	Endemic species impact (% global)	Relative impact/mass consumed	Population (2010)	Per capita impact rank	Embedded soy (tonnes)	Soy consumption (% global total)
**122** Honduras	HND	Central America		0.0	0.54	8194778	115	3055.983	0.0
**123** Nepal	NPL	South Asia	NYDF	0.0	0.35	27023137	132	4296.787	0.0
**124** Mongolia	MNG	East Asia	NYDF	0.0	0.32	2712650	97	4674.436	0.0
**125** El Salvador	SLV	Central America		0.0	0.39	6164626	111	3534.69	0.0
**126** Madagascar	MDG	Eastern Africa		0.0	0.31	21151640	133	3718.987	0.0
**127** Ethiopia	ETH	Eastern Africa	NYDF	0.0	0.29	87702670	140	3783.082	0.0
**128** Nicaragua	NIC	Central America		0.0	0.45	5737723	116	2202.016	0.0
**129** Brunei Darussalam	BRN	Southeast Asia		0.0	0.34	388662	53	2689.414	0.0
**130** Uganda	UGA	Eastern Africa		0.0	0.35	33915133	137	2197.883	0.0
**131** Namibia	NAM	South African Customs Union		0.0	0.50	2173170	105	1488.341	0.0
**132** Zambia	ZMB	Eastern Africa		0.0	0.42	13850033	134	1711.326	0.0
**133** Rest of North America	XNA	North America		0.0	0.37	128303	31	1774.698	0.0
**134** Botswana	BWA	South African Customs Union		0.0	0.34	2014866	113	1275.246	0.0
**135** Rest of South African Customs Union	XSC	South African Customs Union		0.0	0.52	3243394	127	727.0659	0.0
**136** Burkina Faso	BFA	Western Africa	NYDF	0.0	0.32	15605217	136	1166.836	0.0
**137** Malawi	MWI	Eastern Africa		0.0	0.36	15167095	138	771.4063	0.0
**138** Rest of Central America	XCA	Central America		0.0	0.49	321608	94	379.6128	0.0
**139** Rwanda	RWA	Eastern Africa		0.0	0.34	10246842	139	418.7721	0.0
**140** Rest of the World	XTW	Rest of World		0.0	0.49	7810	66	27.7314	0.0

Notes
1) NYDF is New York Declaration on Forests; ADF is Amsterdam Declaration on Forests.
2) [NYDF] indicates that subnational, but not national-level, government signatories
3) In addition to the countries indicated in the table above, the European Union, Democratic Republic of the Congo, Liberia and Guyana all signed the declaration.

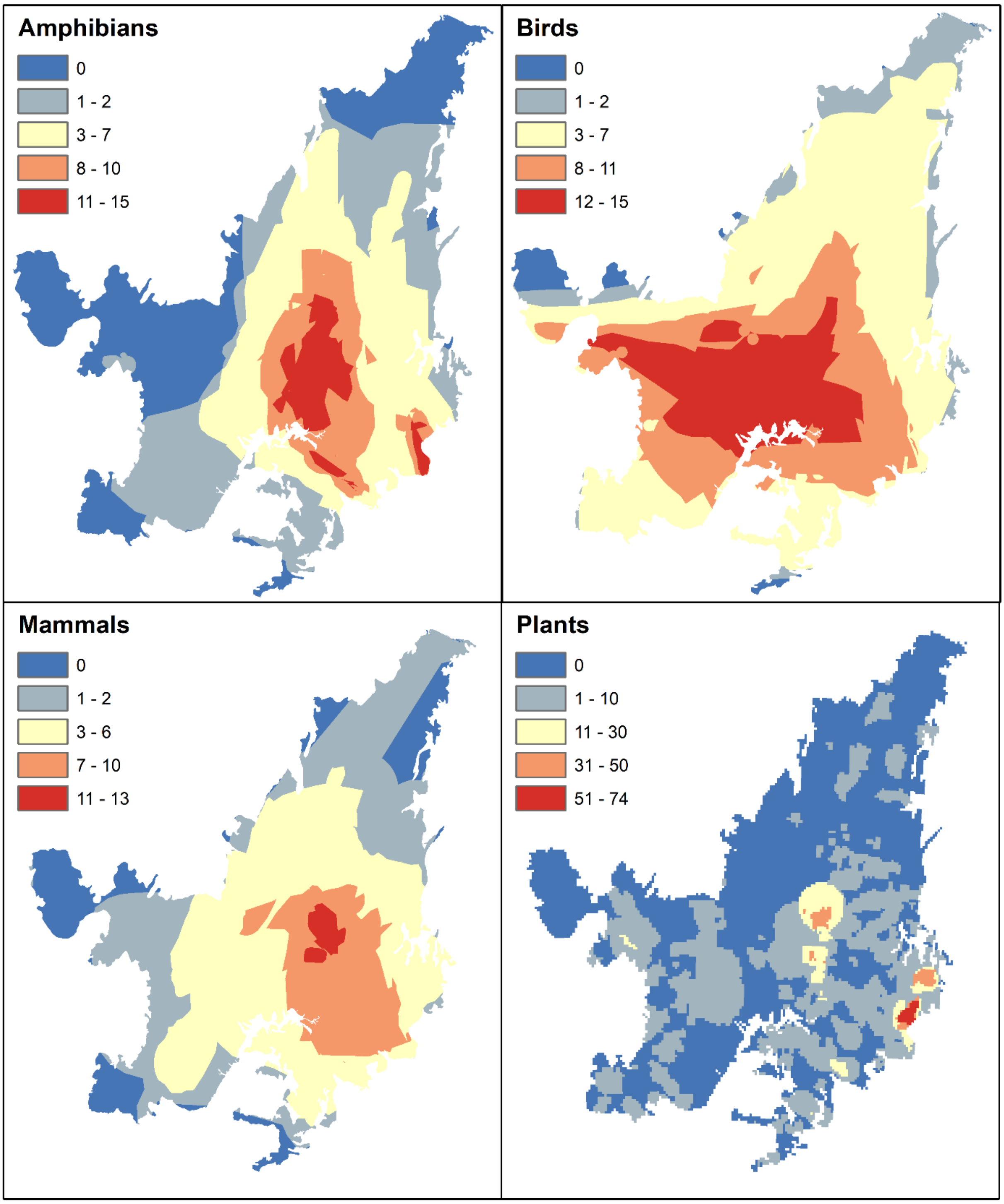

Figure S1. *Richness of endemic and near endemic species used in this study. Maps are based on individual species ranges (extent of occurrence) for endemic and near endemic species (see methods). Table S4 lists the species included.*

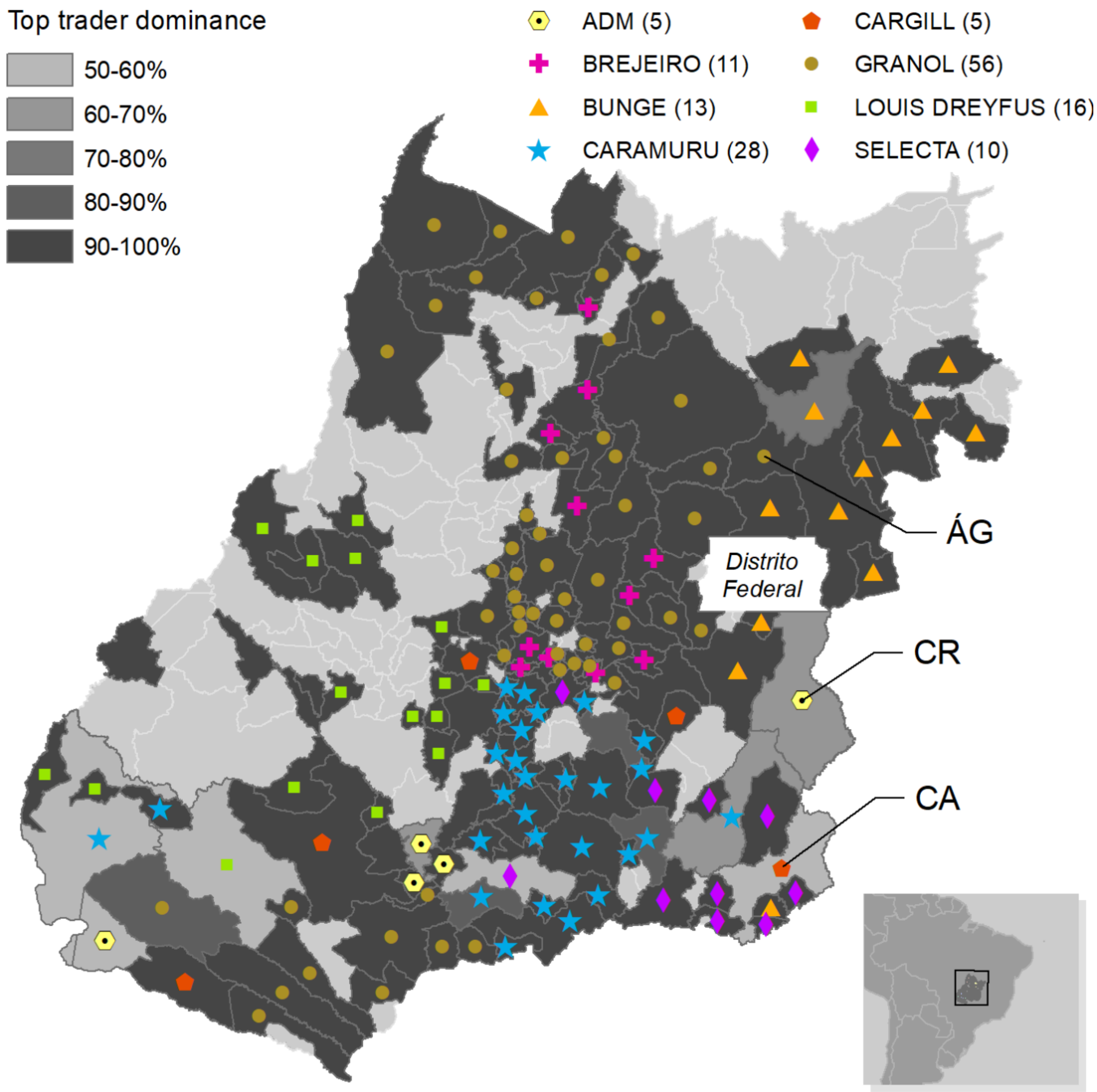

Figure S2. *Exporter dominance at local scales within Goiás state. The export of soy from within each municipality in Goiás tends to be dominated by a single trader with the most dominant trader (coloured symbols) within each municipality accounting for 45-100% of exports (mean=97%) in 2011. Areas that did not produce soy for export in 2011 are in light grey. Labels highlight the three municipalities that experienced the greatest losses of endemic biodiversity between 2000 and 2010: Cristalina, CR; Catalão, CA; and Água Fria de Goiás, ÁG.*

Table S2. *Traders exporting from the state of Goiás, ranked by weight of soy (soy equivalents) traded in 2011. Data from* *Trase* (2).

Trader	Export (soy equivalents)	Percentage of exports
CARAMURU	664,203	18.1%
CARGILL	548,292	15.0%
ADM	513,861	14.0%
LOUIS DREYFUS	497,538	13.6%
BUNGE	329,081	9.0%
GRANOL	327,473	8.9%
MULTIGRAIN	171,389	4.7%
BP BIOENERGIA	103,323	2.8%
SELECTA	101,031	2.8%
CEREAL	81,166	2.2%
ABC INDUSTRIA	65,038	1.8%
BREJEIRO	60,316	1.6%
AGREX	38,600	1.1%
COOP CENTRAL DOS PRODUTORES RURAIS DE MINAS GERAIS	25,769	0.7%
MAEDA AGROINDUSTRIAL	21,142	0.6%
NIDERA	16,633	0.5%
COMPANHIA BRASILEIRA DE DISTRIBUICAO	11,645	0.3%
SIDERURGIA SANTO ANTONIO	10,456	0.3%
ARANTES ALIMENTOSEM RECUPERACAO JUDICIAL	9,973	0.3%
GLENCORE	8,862	0.2%
PRODUQUIMICA INDUSTRIA E COMERCIO	7,185	0.2%
GIRASSOL COMERCIO DE CEREAIS	6,162	0.2%
CORRECTA	5,063	0.1%
GUARANI	4,982	0.1%
NOBLE	4,453	0.1%
QUALY MARCAS COMERCIO E EXPORTACAO DE CEREAIS	3,969	0.1%
MANGELS INDUSTRIA E COMERCIO	3,430	0.1%
COAMO	3,093	0.1%
MITSUBISHI CORPORATION DO BRASIL	3,000	0.1%
SINA	2,062	0.1%
MGT DO BRASIL	2,054	0.1%
OLFAR	1,663	0.0%
GRANITOS MOREDO	1,614	0.0%
NATURALLE AGRO MERCANTIL	1,460	0.0%
BRESPEL COMPANHIA INDUSTRIAL BRASIL ESPANHA	1,322	0.0%
AMAGGI	896	0.0%
SERGIO GUIMARAES GARCIA	535	0.0%
CALIMAN AGRICOLA-EM RECUPERACAO JUDICIAL	533	0.0%
FAUSTO VINICIUS DE GUIMARAES GARCIA	530	0.0%
TIG - TRADING INTERNATIONAL GRANITE	423	0.0%
RONAN BARBOSA GARCIA JUNIOR	300	0.0%
[Domestic Consumption]	[4,040,404]	

Table S3. *Top ten exporters in 2011 by weight for the seven countries that are signatories to the Amsterdam Declaration (export from Brazilian Cerrado to country of first import). Data from* *Trase* (2).

Denmark		France		Germany		Italy		Netherlands		Norway		United Kingdom	
Exporter	***Imports***	***Exporter***	***Imports***	***Exporter***	***Imports***	***Exporter***	***Imports***	***Exporter***	***Imports***	***Exporter***	***Imports***	***Exporter***	***Imports***
Amaggi	*99.0%*	*Louis Dreyfus*	*23.1%*	*Bunge*	*58.1%*	*Amaggi*	*53.9%*	*ADM*	*16.6%*	*Amaggi*	*100.0%*	*Cargill*	*64.5%*
ADM	*1.0%*	*Cargill*	*22.9%*	*ADM*	*17.2%*	*Amaggi & LD Commodities*	*17.5%*	*Cargill*	*13.5%*	-	-	*Caramuru*	*17.9%*
-	-	*Bunge*	*13.2%*	*Granol*	*9.5%*	*Multigrain*	*12.8%*	*Granol*	*12.6%*	-	-	*Louis Dreyfus*	*16.1%*
-	-	*ADM*	*9.2%*	*Brejeiro*	*4.2%*	*Louis Dreyfus*	*9.9%*	*Caramuru*	*12.6%*	-	-	*Bunge*	*1.5%*
-	-	*Granol*	*8.2%*	*Multigrain*	*2.5%*	*Glencore*	*3.2%*	*Amaggi*	*9.7%*	-	-	-	-
-	-	*Vanguarda Do Brasil*	*5.4%*	*Noble*	*1.9%*	*Cargill*	*2.8%*	*Agrex*	*9.6%*	-	-	-	-
-	-	*Intercoop*	*3.4%*	*Cargill*	*1.5%*	-	-	*Louis Dreyfus*	*7.5%*	-	-	-	-
-	-	*Amaggi*	*3.1%*	*Selecta*	*1.3%*	-	-	*Selecta*	*5.4%*	-	-	-	-
-	-	*Cereal*	*2.7%*	*Amaggi & LD Commodities*	*1.3%*	-	-	*ABC Industria*	*4.3%*	-	-	-	-
-	-	*Correcta*	*2.2%*	*BP Bioenergia*	*1.0%*	-	-	*Bunge*	*2.8%*	-	-	-	-

Table S4. *Species included in analyses. Columns detail, for each species, the proportion of their range that falls within the Cerrado study area, the original extent of suitable habitat (estimated from pre-industrial land cover), extent of suitable habitat in the years 2000, 2010, 2012 and 2014. The corresponding probabilities of persistence are also calculated and shown for the same years. Mean extent of suitable habitat in 2000 was 15,516 km² for amphibians, 16,728 km² for birds, 32,192 km² for mammals, and 10,104 km² for plants.*

Species	Proportion global range within Cerrado	Extent of Suitable Habitat (km2)					Probability of persistence				
		Original	yr 2000	yr 2010	yr 2012	yr 2014	Original	yr 2000	yr 2010	yr 2012	yr 2014
**Amphibians**											
Allobates goianus	1.00	673	130	129	128	127	1	0.663	0.662	0.660	0.660
Ameerega flavopicta	0.72	161,716	18,350	16,114	15,280	14,592	1	0.580	0.562	0.554	0.548
Barycholos ternetzi	0.98	130,006	9,602	6,206	5,680	5,461	1	0.521	0.467	0.457	0.453
Bokermannohyla martinsi	0.72	5,377	5,358	5,358	5,358	5,157	1	0.999	0.999	0.999	0.990
Bokermannohyla pseudopseudis	0.99	1,870	317	296	258	258	1	0.641	0.631	0.609	0.609
Bokermannohyla sazimai	1.00	7,978	1,755	1,703	1,703	1,697	1	0.685	0.680	0.680	0.679
Chiasmocleis centralis	1.00	16,766	635	313	304	304	1	0.441	0.370	0.367	0.367
Dendropsophus jimi	0.75	18,360	2,742	2,717	2,713	2,599	1	0.622	0.620	0.620	0.613
Elachistocleis piauiensis	0.80	15,212	11,774	10,890	10,863	9,702	1	0.938	0.920	0.919	0.894
Hypsiboas cipoensis	0.82	5,317	5,009	5,009	5,009	4,830	1	0.985	0.985	0.985	0.976
Hypsiboas goianus	0.98	32,147	2,349	2,049	1,864	1,794	1	0.520	0.502	0.491	0.486
Hypsiboas phaeopleura	1.00	85	43	3	2	2	1	0.840	0.448	0.391	0.391
Ischnocnema penaxavantinho	0.96	11,012	1,631	1,558	1,451	1,402	1	0.620	0.613	0.602	0.597
Leptodactylus camaquara	0.85	4,178	4,178	4,178	4,178	4,008	1	1.000	1.000	1.000	0.990
Leptodactylus sertanejo	0.90	72,999	8,528	7,252	6,688	6,231	1	0.585	0.561	0.550	0.541
Odontophrynus cultripes	0.82	82,498	19,896	19,502	19,279	18,556	1	0.701	0.697	0.695	0.689
Odontophrynus salvatori	1.00	11,679	2,100	1,795	1,706	1,705	1	0.651	0.626	0.618	0.618
Oreobates crepitans	0.75	1,140	46	38	38	38	1	0.448	0.428	0.426	0.426
Phasmahyla jandaia	0.70	5,101	4,860	4,859	4,859	4,673	1	0.988	0.988	0.988	0.978
Phyllomedusa centralis	1.00	197	10	10	10	10	1	0.474	0.470	0.470	0.470
Physalaemus deimaticus	0.97	460	460	460	460	449	1	1.000	1.000	1.000	0.994
Physalaemus evangelistai	0.77	110	110	109	109	101	1	0.999	0.999	0.999	0.979
Pristimantis crepitans	0.75	1,140	46	38	38	38	1	0.448	0.428	0.426	0.426
Pristimantis dundeei	0.82	1,037	151	135	129	103	1	0.617	0.600	0.594	0.561
Proceratophrys cururu	1.00	407	407	407	407	398	1	1.000	1.000	1.000	0.994
Proceratophrys goyana	1.00	2,466	227	190	132	132	1	0.551	0.527	0.481	0.481
Pseudis bolbodactyla	0.75	119,339	18,939	17,421	16,069	15,704	1	0.631	0.618	0.606	0.602
Pseudis fusca	0.77	6,411	4,208	4,176	4,149	2,488	1	0.900	0.898	0.897	0.789
Pseudis tocantins	1.00	32,171	8,234	7,994	7,944	7,907	1	0.711	0.706	0.705	0.704
Pseudopaludicola mineira	0.98	432	432	432	432	420	1	1.000	1.000	1.000	0.993
Pseudopaludicola saltica	0.75	218,915	179,149	174,992	173,580	159,879	1	0.951	0.946	0.944	0.924
Pseudopaludicola ternetzi	0.90	45,730	40,349	39,269	38,276	35,966	1	0.969	0.963	0.956	0.942
Rhinella cerradensis	0.92	59,092	3,310	2,803	2,254	2,205	1	0.486	0.467	0.442	0.439
Rhinella rubescens	0.77	207,207	116,928	113,666	111,680	92,554	1	0.867	0.861	0.857	0.818
Rhinella veredas	0.99	10,324	1,895	1,836	1,185	1,117	1	0.655	0.649	0.582	0.574
Scinax cabralensis	1.00	1	1	0	0	0	1	1.000	0.863	0.863	0.863
Scinax canastrensis	1.00	1,436	1,430	1,391	1,391	1,391	1	0.999	0.992	0.992	0.992

Species	Proportion global range within Cerrado	Extent of Suitable Habitat (km2) Original	yr 2000	yr 2010	yr 2012	yr 2014	Probability of persistence Original	yr 2000	yr 2010	yr 2012	yr 2014
Scinax centralis	1.00	9,931	867	735	609	580	1	0.544	0.522	0.498	0.492
Scinax constrictus	1.00	11,498	333	126	62	56	1	0.412	0.324	0.271	0.264
Scinax maracaya	1.00	746	745	711	711	711	1	1.000	0.988	0.988	0.988
Scinax pinima	0.99	402	402	402	402	394	1	1.000	1.000	1.000	0.995
**Birds**											
Alipiopsitta xanthops	0.80	170,432	13,950	10,444	8,981	8,405	1	0.535	0.498	0.479	0.471
Antilophia galeata	0.77	88,764	8,053	5,812	5,121	4,945	1	0.549	0.506	0.490	0.486
Asthenes luizae	0.89	5,068	5,012	5,012	5,012	4,843	1	0.997	0.997	0.997	0.989
Basileuterus hypoleucus	0.71	142,249	13,937	8,213	6,634	6,481	1	0.559	0.490	0.465	0.462
Basileuterus leucophrys	0.86	102,157	4,172	2,606	2,385	2,290	1	0.450	0.400	0.391	0.387
Celeus obrieni	0.93	111,725	11,279	8,604	7,875	7,487	1	0.564	0.527	0.515	0.509
Cercomacra ferdinandi	0.81	11,622	10,656	10,571	10,570	10,550	1	0.979	0.977	0.977	0.976
Columbina cyanopis	0.72	159,407	4,226	1,590	1,087	1,020	1	0.403	0.316	0.287	0.283
Geositta poeciloptera	0.71	183,779	16,617	13,258	12,292	11,771	1	0.548	0.518	0.509	0.503
Knipolegus franciscanus	0.92	3,486	11	7	6	6	1	0.235	0.215	0.201	0.201
Malacoptila minor	0.72	56,977	4,729	952	913	889	1	0.537	0.360	0.356	0.353
Paroaria baeri	0.98	24,481	14,407	13,475	13,376	13,323	1	0.876	0.861	0.860	0.859
Phyllomyias reiseri	0.94	11,907	445	216	210	210	1	0.440	0.367	0.364	0.364
Phylloscartes roquettei	0.72	33,207	1,520	1,378	1,351	1,165	1	0.463	0.451	0.449	0.433
Poospiza cinerea	0.75	114,639	15,605	13,847	12,872	12,356	1	0.607	0.590	0.579	0.573
Porphyrospiza caerulescens	0.83	108,934	11,233	9,156	7,897	7,195	1	0.567	0.538	0.519	0.507
Scytalopus novacapitalis	1.00	3,284	5	4	2	2	1	0.193	0.189	0.146	0.146
Suiriri islerorum	0.85	126,717	8,616	2,742	2,324	2,093	1	0.511	0.384	0.368	0.358
Syndactyla dimidiata	0.72	156,229	13,751	6,689	5,232	5,042	1	0.545	0.455	0.428	0.424
Taoniscus nanus	0.75	146,440	9,844	7,603	6,662	6,083	1	0.509	0.477	0.462	0.451
Uropelia campestris	0.76	250,769	124,308	118,027	115,655	97,279	1	0.839	0.828	0.824	0.789
**Mammals**											
Callithrix penicillata	0.84	375,214	38,716	32,437	30,711	29,555	1	0.567	0.542	0.535	0.530
Calomys tocantinsi	0.90	46,245	20,456	17,021	16,556	16,467	1	0.816	0.779	0.774	0.772
Carterodon sulcidens	0.99	57,621	6,007	5,696	5,126	4,906	1	0.568	0.561	0.546	0.540
Cerradomys marinhus	1.00	3,642	82	82	-	-	1	0.388	0.388	0.000	0.000
Chrysocyon brachyurus	0.42	713,629	689,081	674,501	671,146	652,636	1	0.991	0.986	0.985	0.978
Euryoryzomys lamia	0.95	13,034	735	608	480	457	1	0.487	0.465	0.438	0.433
Galea flavidens	0.98	24,789	6,537	6,350	6,268	5,934	1	0.717	0.711	0.709	0.699
Kunsia fronto	1.00	80	7	4	2	2	1	0.546	0.464	0.395	0.395
Lonchophylla dekeyseri	1.00	2,410	594	544	503	502	1	0.705	0.689	0.676	0.676
Microakodontomys transitorius	1.00	744	356	335	324	324	1	0.832	0.819	0.813	0.813
Monodelphis kunsi	0.96	173,364	12,865	8,494	7,853	7,549	1	0.522	0.470	0.461	0.457
Myrmecophaga tridactyla	0.17	807,337	139,810	113,799	104,252	100,322	1	0.645	0.613	0.599	0.594
Oecomys cleberi	1.00	23	8	8	8	8	1	0.778	0.769	0.769	0.769
Oligoryzomys moojeni	1.00	24,478	4,341	3,181	2,448	2,058	1	0.649	0.600	0.562	0.538
Oxymycterus delator	1.00	886	884	857	857	857	1	0.999	0.992	0.992	0.992

Species	Proportion global range within Cerrado	Extent of Suitable Habitat (km2) Original	yr 2000	yr 2010	yr 2012	yr 2014	Probability of persistence Original	yr 2000	yr 2010	yr 2012	yr 2014
Oxymycterus roberti	0.81	59,531	25,167	24,392	23,654	18,142	1	0.806	0.800	0.794	0.743
Pseudalopex vetulus	0.82	338,238	323,184	314,223	311,564	301,824	1	0.989	0.982	0.980	0.972
Rhipidomys macrurus	0.85	418,558	51,476	38,103	35,577	34,346	1	0.592	0.549	0.540	0.535
Thalpomys cerradensis	0.96	82,712	6,633	4,914	3,818	3,681	1	0.532	0.494	0.464	0.459
Thalpomys lasiotis	0.85	77,924	12,880	12,249	11,112	10,573	1	0.638	0.630	0.615	0.607
Thylamys velutinus	0.89	110,131	6,719	5,770	5,260	5,160	1	0.497	0.478	0.467	0.465
Thyroptera devivoi	1.00	10,988	38	37	37	-	1	0.242	0.241	0.241	0.000
Trinomys moojeni	0.98	927	927	927	927	924	1	1.000	1.000	1.000	0.999
Wiedomys cerradensis	1.00	776	1	1	-	-	1	0.189	0.189	0.000	0.000
**Plants**											
Acritopappus irwinii	1.00	14,218	12,251	11,095	10,694	8,502	1	0.963	0.940	0.931	0.879
Actinocephalus cipoensis	1.00	703	370	370	370	362	1	0.852	0.852	0.852	0.847
Actinocephalus claussenianus	1.00	3,703	2,556	2,524	2,456	2,392	1	0.911	0.909	0.902	0.896
Aeschynomene laca-buendiana	1.00	589	588	587	511	315	1	1.000	0.999	0.965	0.855
Agalinis nana	1.00	4,624	3,135	3,125	3,115	3,105	1	0.907	0.907	0.906	0.905
Aiouea bracteata	1.00	7,128	2,961	2,921	2,818	2,716	1	0.803	0.800	0.793	0.786
Alcantarea duarteana	0.92	11,272	11,075	10,864	10,679	8,101	1	0.996	0.991	0.987	0.921
Aldama filifolia	1.00	35,645	29,276	28,161	27,103	26,899	1	0.952	0.943	0.934	0.932
Aldama goyazii	0.95	18,682	13,147	12,509	11,670	4,532	1	0.916	0.905	0.889	0.702
Aldama linearifolia	0.86	7,011	1,159	918	652	518	1	0.638	0.602	0.552	0.521
Aldama vernonioides	1.00	13,669	6,166	5,740	5,201	5,068	1	0.820	0.805	0.785	0.780
Alstroemeria brasiliensis	1.00	11,679	8,289	7,695	7,624	4,426	1	0.918	0.901	0.899	0.785
Alstroemeria orchidioides	1.00	2,724	1,840	1,460	1,313	1,200	1	0.907	0.856	0.833	0.815
Alstroemeria penduliflora	1.00	12,796	10,315	8,800	8,483	6,032	1	0.948	0.911	0.902	0.829
Alternanthera decurrens	1.00	724	575	551	393	376	1	0.944	0.934	0.858	0.849
Alternanthera januarensis	1.00	952	952	952	950	902	1	1.000	1.000	1.000	0.987
Altoparadisium chapadense	1.00	1,793	1,633	1,506	1,501	1,493	1	0.977	0.957	0.957	0.955
Anemia trichorhiza	1.00	67,861	50,539	45,039	42,597	41,259	1	0.929	0.903	0.890	0.883
Anemopaegma arvense	0.75	356,701	215,864	190,618	175,260	134,414	1	0.882	0.855	0.837	0.783
Anemopaegma mirabile	0.73	6,301	5,915	5,811	5,674	5,568	1	0.984	0.980	0.974	0.970
Angelonia alternifolia	1.00	845	830	749	749	736	1	0.996	0.970	0.970	0.966
Anteremanthus hatschbachii	1.00	10,750	9,491	9,152	8,232	6,002	1	0.969	0.961	0.935	0.864
Arrojadoa eriocaulis	0.97	5,795	5,156	4,971	4,476	3,376	1	0.971	0.962	0.937	0.874
Arthrocereus rondonianus	1.00	11,271	10,837	10,467	10,165	7,909	1	0.990	0.982	0.975	0.915
Arundinella deppeana	0.84	5,371	3,884	3,807	3,670	883	1	0.922	0.918	0.909	0.637
Aspilia almasensis	0.85	6,141	5,510	5,324	4,758	3,463	1	0.973	0.965	0.938	0.867
Aspilia cylindrocephala	1.00	18,511	12,017	10,940	10,391	7,828	1	0.898	0.877	0.866	0.806
Aspilia diamantinae	1.00	6,933	6,816	6,609	6,427	5,719	1	0.996	0.988	0.981	0.953
Aspilia diffusiflora	0.84	12,438	12,127	11,869	11,656	8,488	1	0.994	0.988	0.984	0.909
Aspilia eglerii	1.00	6,816	6,699	6,491	6,310	5,605	1	0.996	0.988	0.981	0.952
Aspilia espinhacensis	1.00	11,427	9,677	9,080	8,503	6,818	1	0.959	0.944	0.929	0.879
Aspilia jugata	1.00	1,762	1,756	1,742	1,739	1,696	1	0.999	0.997	0.997	0.990
Aspilia pereirae	1.00	20,311	12,918	10,642	9,877	8,409	1	0.893	0.851	0.835	0.802

Species	Proportion global range within Cerrado	Extent of Suitable Habitat (km2)					Probability of persistence				
		Original	yr 2000	yr 2010	yr 2012	yr 2014	Original	yr 2000	yr 2010	yr 2012	yr 2014
Aspilia prostrata	1.00	1,396	1,036	1,003	978	703	1	0.928	0.921	0.915	0.842
Astraea cincta	0.98	47,853	26,509	24,364	21,622	10,754	1	0.863	0.845	0.820	0.689
Attalea barreirensis	1.00	20,218	17,099	15,754	15,058	14,182	1	0.959	0.940	0.929	0.915
Attalea brasiliensis	1.00	8,899	6,441	5,063	4,737	4,632	1	0.922	0.868	0.854	0.849
Axonopus hydrolithicus	1.00	12,643	8,685	7,825	7,626	7,586	1	0.910	0.887	0.881	0.880
Axonopus uninodis	0.91	8,298	5,769	4,471	4,127	3,869	1	0.913	0.857	0.840	0.826
Baccharis elliptica	0.87	14,891	14,392	14,107	13,862	9,581	1	0.992	0.987	0.982	0.896
Baccharis pseudoalpestris	0.90	37,957	33,178	31,173	28,976	21,178	1	0.967	0.952	0.935	0.864
Banisteriopsis cipoensis	0.80	52,811	42,115	41,174	40,140	37,884	1	0.945	0.940	0.934	0.920
Banisteriopsis hatschbachii	1.00	35,654	29,195	28,425	27,529	27,377	1	0.951	0.945	0.937	0.936
Banisteriopsis hirsuta	1.00	13,477	11,753	11,368	10,621	10,449	1	0.966	0.958	0.942	0.938
Barbacenia glutinosa	0.83	708	685	683	683	583	1	0.992	0.991	0.991	0.953
Barbacenia longiscapa	1.00	1,883	1,875	1,873	1,870	1,763	1	0.999	0.999	0.998	0.984
Barbacenia paranaensis	0.71	3,066	694	412	403	398	1	0.690	0.605	0.602	0.600
Barbacenia pungens	1.00	702	701	701	699	689	1	0.999	0.999	0.999	0.995
Barbacenia riparia	1.00	10,750	9,491	9,152	8,232	6,002	1	0.969	0.961	0.935	0.864
Barbacenia rodriguesii	1.00	704	703	703	703	560	1	1.000	1.000	1.000	0.944
Barbacenia spiralis	1.00	6,816	6,699	6,491	6,310	5,605	1	0.996	0.988	0.981	0.952
Blechnum heringeri	1.00	12,938	7,811	6,913	6,460	5,098	1	0.881	0.855	0.841	0.792
Brasilicereus markgrafii	1.00	6,850	5,994	5,782	5,171	3,911	1	0.967	0.958	0.932	0.869
Bromelia braunii	1.00	482	471	406	406	405	1	0.994	0.958	0.958	0.957
Butia capitata	1.00	32,721	24,879	23,595	21,623	13,767	1	0.934	0.922	0.902	0.805
Butia leptospatha	0.94	18,761	3,853	3,015	1,951	1,460	1	0.673	0.633	0.568	0.528
Butia purpurascens	0.94	10,790	6,703	6,352	5,801	1,033	1	0.888	0.876	0.856	0.556
Calea abbreviata	1.00	6,081	5,351	5,320	5,098	5,073	1	0.969	0.967	0.957	0.956
Calea brittoniana	1.00	4,045	2,815	2,806	2,802	2,801	1	0.913	0.913	0.912	0.912
Calliandra carrascana	1.00	2,386	2,172	2,119	1,806	1,676	1	0.977	0.971	0.933	0.916
Camarea humifusa	1.00	5,253	4,508	4,484	4,254	4,234	1	0.963	0.961	0.949	0.948
Camarea linearifolia	1.00	4,162	3,055	3,046	3,044	3,033	1	0.926	0.925	0.925	0.924
Cambessedesia atropurpurea	0.86	9,431	8,079	7,877	7,450	7,381	1	0.962	0.956	0.943	0.941
Canastra lanceolata	0.93	6,141	3,919	3,879	3,811	3,746	1	0.894	0.892	0.888	0.884
Cattleya walkeriana	0.92	45,674	30,124	27,306	25,596	14,857	1	0.901	0.879	0.865	0.755
Cereus mirabella	0.94	34,261	22,923	21,108	18,570	15,086	1	0.904	0.886	0.858	0.815
Chamaecrista fodinarum	0.75	7,326	6,337	6,051	5,488	3,886	1	0.964	0.953	0.930	0.853
Chamaecrista stillifera	1.00	23,919	21,952	21,063	19,724	15,013	1	0.979	0.969	0.953	0.890
Chamaecrista tephrosiifolia	1.00	11,107	9,811	9,454	8,540	6,302	1	0.969	0.961	0.936	0.868
Chamaecrista ulmea	1.00	7,326	6,337	6,051	5,488	3,886	1	0.964	0.953	0.930	0.853
Chresta souzae	1.00	36,603	29,704	28,903	28,004	27,852	1	0.949	0.943	0.935	0.934
Chromolaena arrayana	1.00	12,643	8,685	7,825	7,626	7,586	1	0.910	0.887	0.881	0.880
Chromolaena costatipes	1.00	4,162	3,055	3,046	3,044	3,033	1	0.926	0.925	0.925	0.924
Cipocereus bradei	1.00	9,033	8,526	8,062	7,893	5,817	1	0.986	0.972	0.967	0.896
Cipocereus crassisepalus	0.72	8,252	7,434	6,999	6,472	5,635	1	0.974	0.960	0.941	0.909
Cissus inundata	1.00	8,235	6,553	6,261	5,734	4,124	1	0.944	0.934	0.914	0.841
Cleistes aphylla	0.94	36,910	31,333	28,782	27,845	25,438	1	0.960	0.940	0.932	0.911

Species	Proportion global range within Cerrado	Extent of Suitable Habitat (km2)					Probability of persistence				
		Original	yr 2000	yr 2010	yr 2012	yr 2014	Original	yr 2000	yr 2010	yr 2012	yr 2014
Comanthera elegans	0.86	15,900	14,676	14,192	13,416	10,638	1	0.980	0.972	0.958	0.904
Cuphea cuiabensis	0.92	20,657	11,039	10,122	9,497	9,365	1	0.855	0.837	0.823	0.821
Cuphea rubro-virens	1.00	1,414	1,412	1,345	1,214	876	1	1.000	0.988	0.963	0.887
Cuphea teleandra	1.00	7,326	6,337	6,051	5,488	3,886	1	0.964	0.953	0.930	0.853
Cyanocephalus caprariifolius	1.00	15,311	9,940	7,966	6,961	5,190	1	0.898	0.849	0.821	0.763
Cyanocephalus digitatus	1.00	40,655	33,574	32,559	31,321	31,099	1	0.953	0.946	0.937	0.935
Cyanocephalus tagetifolius	1.00	4,778	4,366	4,307	4,129	4,106	1	0.978	0.974	0.964	0.963
Cycnoches pentadactylum	1.00	9,236	7,050	7,029	6,767	6,705	1	0.935	0.934	0.925	0.923
Cyrtopodium caiapoense	1.00	39,841	28,015	24,544	23,258	21,494	1	0.916	0.886	0.874	0.857
Cyrtopodium hatschbachii	0.88	17,678	11,145	10,381	9,678	1,091	1	0.891	0.875	0.860	0.498
Cyrtopodium latifolium	1.00	8,183	5,739	4,361	4,049	3,944	1	0.915	0.854	0.839	0.833
Cyrtopodium linearifolium	1.00	35,654	29,195	28,425	27,529	27,377	1	0.951	0.945	0.937	0.936
Cyrtopodium lissochiloides	0.89	19,425	15,931	14,322	13,476	12,653	1	0.952	0.927	0.913	0.898
Cyrtopodium triste	0.82	8,183	5,739	4,361	4,049	3,944	1	0.915	0.854	0.839	0.833
Dichorisandra glaziovii	0.84	9,923	8,924	8,680	7,818	5,734	1	0.974	0.967	0.942	0.872
Digitaria neesiana	0.86	43,714	25,052	20,179	17,919	16,195	1	0.870	0.824	0.800	0.780
Dimerostemma annuum	1.00	18,063	7,747	4,667	3,111	2,928	1	0.809	0.713	0.644	0.635
Dimerostemma grazielae	1.00	53,236	42,837	40,233	38,396	37,343	1	0.947	0.932	0.922	0.915
Dimorphandra wilsonii	0.93	3,262	1,543	1,423	1,376	579	1	0.829	0.813	0.806	0.649
Dioscorea asperula	1.00	14,385	8,868	6,942	6,451	5,753	1	0.886	0.833	0.818	0.795
Diplusodon aggregatifolius	1.00	3,892	3,711	3,518	3,209	2,226	1	0.988	0.975	0.953	0.870
Diplusodon ericoides	1.00	5,846	4,683	4,534	4,176	4,119	1	0.946	0.938	0.919	0.916
Diplusodon glaziovii	1.00	702	702	702	702	672	1	1.000	1.000	1.000	0.989
Diplusodon gracilis	1.00	1,085	1,080	1,074	1,069	1,064	1	0.999	0.997	0.996	0.995
Diplusodon hatschbachii	1.00	8,714	7,423	7,248	6,844	6,781	1	0.961	0.955	0.941	0.939
Diplusodon minasensis	0.99	7,292	7,174	6,966	6,785	6,008	1	0.996	0.989	0.982	0.953
Diplusodon ovatus	0.98	23,192	13,108	9,982	9,321	5,467	1	0.867	0.810	0.796	0.697
Diplusodon panniculatus	1.00	1,062	777	689	622	535	1	0.925	0.898	0.875	0.843
Diplusodon retroimbricatus	1.00	9,032	7,872	7,476	7,002	6,665	1	0.966	0.954	0.938	0.927
Discocactus catingicola	0.93	36,191	28,231	26,328	22,769	21,765	1	0.940	0.924	0.891	0.881
Discocactus horstii	1.00	5,552	4,922	4,737	4,247	3,147	1	0.970	0.961	0.935	0.868
Discocactus pseudoinsignis	1.00	6,850	5,994	5,782	5,171	3,911	1	0.967	0.958	0.932	0.869
Disynaphia praeficta	0.85	14,805	14,081	13,681	13,437	9,873	1	0.988	0.980	0.976	0.904
Ditassa auriflora	1.00	3,066	2,906	2,715	2,433	1,594	1	0.987	0.970	0.944	0.849
Ditassa cordeiroana	1.00	13,829	12,388	11,932	10,825	8,236	1	0.973	0.964	0.941	0.878
Dorstenia conceptionis	0.87	5,716	3,433	3,049	2,939	287	1	0.880	0.855	0.847	0.473
Drosera graomogolensis	1.00	10,869	9,628	9,290	8,393	6,154	1	0.970	0.962	0.937	0.867
Echinocoryne echinocephala	1.00	47,656	38,044	35,605	34,130	33,206	1	0.945	0.930	0.920	0.914
Encholirium disjunctum	1.00	2,511	1,585	1,383	1,343	1,302	1	0.891	0.862	0.855	0.848
Encholirium irwinii	1.00	7,326	6,337	6,051	5,488	3,886	1	0.964	0.953	0.930	0.853
Encholirium luxor	0.96	19,473	16,822	16,162	13,875	12,735	1	0.964	0.954	0.919	0.899
Encholirium pedicellatum	1.00	6,816	6,699	6,491	6,310	5,605	1	0.996	0.988	0.981	0.952
Encholirium scrutor	0.94	1,539	1,529	1,527	1,524	1,413	1	0.998	0.998	0.997	0.979
Eptesicus diminutus	0.20	3,444	950	938	883	868	1	0.725	0.722	0.711	0.708

Species	Proportion global range within Cerrado	Extent of Suitable Habitat (km2) Original	yr 2000	yr 2010	yr 2012	yr 2014	Probability of persistence Original	yr 2000	yr 2010	yr 2012	yr 2014
Eremanthus argenteus	1.00	35,654	29,195	28,425	27,529	27,377	1	0.951	0.945	0.937	0.936
Eriope crassipes subsp. cristalinae	1.00	7,401	5,227	4,244	3,821	2,670	1	0.917	0.870	0.848	0.775
Eriope machrisae	1.00	36,608	29,877	29,013	28,065	27,898	1	0.950	0.944	0.936	0.934
Esenbeckia irwiniana	1.00	14,142	13,036	12,543	11,798	9,491	1	0.980	0.970	0.956	0.905
Eugenia blanda	1.00	6,500	5,618	5,342	4,821	3,419	1	0.964	0.952	0.928	0.852
Euphorbia attastoma	0.81	20,961	18,305	16,974	15,810	11,324	1	0.967	0.949	0.932	0.857
Evolvulus glaziovii	0.84	10,475	10,179	9,938	9,488	7,919	1	0.993	0.987	0.976	0.932
Evolvulus kramerioides	0.80	12,831	7,270	6,370	5,917	4,555	1	0.868	0.839	0.824	0.772
Evolvulus rariflorus	1.00	64,016	48,486	44,517	42,238	41,287	1	0.933	0.913	0.901	0.896
Evolvulus riedelii	0.96	16,721	2,526	2,260	2,133	1,896	1	0.623	0.606	0.598	0.580
Evolvulus stellariifolius	0.88	12,359	11,344	11,097	9,758	8,767	1	0.979	0.973	0.943	0.918
Facheiroa cephaliomelana	0.78	17,699	14,505	14,082	10,903	10,620	1	0.951	0.944	0.886	0.880
Facheiroa cephaliomelana subsp. estevesii	1.00	2,409	1,130	973	931	931	1	0.828	0.797	0.788	0.788
Froelichiella grisea	1.00	1,670	1,379	1,201	1,124	1,109	1	0.953	0.921	0.906	0.903
Gaultheria sleumeriana	0.77	919	139	138	137	40	1	0.623	0.622	0.621	0.455
Gaylussacia centunculifolia	1.00	3,611	2,604	2,570	2,536	1,984	1	0.922	0.918	0.915	0.861
Gouania inornata	0.99	17,940	11,226	9,084	8,310	7,397	1	0.889	0.844	0.825	0.801
Goyazia petraea	0.77	18,230	8,929	8,089	7,576	7,345	1	0.837	0.816	0.803	0.797
Griffinia aracensis	1.00	1,161	224	212	209	106	1	0.663	0.653	0.652	0.550
Griffinia nocturna	1.00	3,353	2,318	2,284	2,185	2,183	1	0.912	0.908	0.899	0.898
Gymnopogon doellii	1.00	8,183	5,739	4,361	4,049	3,944	1	0.915	0.854	0.839	0.833
Gyrostelma oxypetaloides	1.00	1,661	1,166	1,106	1,092	1,083	1	0.915	0.903	0.900	0.899
Habenaria galeandriformis	1.00	1,156	411	406	377	377	1	0.772	0.770	0.756	0.756
Harpalyce parvifolia	1.00	18,629	17,223	16,669	15,528	12,419	1	0.981	0.973	0.956	0.904
Hemipogon abietoides	0.86	938	906	903	901	765	1	0.992	0.991	0.990	0.950
Hemipogon furlanii	0.87	30,487	24,261	22,143	19,326	14,135	1	0.944	0.923	0.892	0.825
Heteropterys aliciae	1.00	14,308	12,836	12,770	11,377	11,080	1	0.973	0.972	0.944	0.938
Hippeastrum goianum	1.00	17,154	11,337	8,796	7,853	7,092	1	0.902	0.846	0.823	0.802
Hippeastrum leucobasis	1.00	13,516	10,759	10,429	9,987	5,701	1	0.945	0.937	0.927	0.806
Hoffmannseggella briegeri	0.82	9,746	9,301	9,085	8,897	7,770	1	0.988	0.983	0.977	0.945
Huberia pirani	0.92	11,272	11,075	10,864	10,679	8,101	1	0.996	0.991	0.987	0.921
Hymenophyllum silveirae	0.74	355	271	269	269	269	1	0.934	0.933	0.933	0.933
Hypenia aristulata	1.00	1,912	1,122	912	903	891	1	0.875	0.831	0.829	0.826
Hypenia crispata	1.00	1,896	1,502	1,157	873	763	1	0.943	0.884	0.824	0.797
Hypenia micrantha	1.00	951	722	685	655	655	1	0.933	0.921	0.911	0.911
Hypenia subrosea	1.00	40,655	33,574	32,559	31,321	31,099	1	0.953	0.946	0.937	0.935
Hyptidendron claussenii	1.00	6,816	6,699	6,491	6,310	5,605	1	0.996	0.988	0.981	0.952
Hyptidendron conspersum	1.00	19,612	15,082	13,870	12,395	11,345	1	0.936	0.917	0.892	0.872
Hyptis alpestris	1.00	7,295	4,705	4,564	4,423	3,655	1	0.896	0.889	0.882	0.841
Hyptis angustifolia	1.00	1,421	1,147	898	662	561	1	0.948	0.892	0.826	0.793
Hyptis arenaria	1.00	9,171	8,472	8,270	8,082	7,944	1	0.980	0.974	0.969	0.965
Hyptis colligata	1.00	5,615	4,637	4,514	4,362	4,343	1	0.953	0.947	0.939	0.938
Hyptis cruciformis	1.00	40,655	33,574	32,559	31,321	31,099	1	0.953	0.946	0.937	0.935
Hyptis frondosa	0.97	23,577	13,960	13,265	12,559	10,133	1	0.877	0.866	0.854	0.810

Species	Proportion global range within Cerrado	Extent of Suitable Habitat (km2) Original	yr 2000	yr 2010	yr 2012	yr 2014	Probability of persistence Original	yr 2000	yr 2010	yr 2012	yr 2014
Hyptis hamatidens	1.00	2,595	1,830	1,529	1,403	935	1	0.916	0.876	0.857	0.775
Hyptis imbricatiformis	1.00	40,655	33,574	32,559	31,321	31,099	1	0.953	0.946	0.937	0.935
Hyptis pachyphylla	1.00	15,394	13,273	12,830	11,972	11,800	1	0.964	0.955	0.939	0.936
Hyptis penaeoides	1.00	35,654	29,195	28,425	27,529	27,377	1	0.951	0.945	0.937	0.936
Ichthyothere elliptica	1.00	20,234	12,876	10,546	9,384	7,278	1	0.893	0.850	0.825	0.774
Ipomoea macedoi	0.79	3,513	2,926	2,854	2,691	433	1	0.955	0.949	0.936	0.593
Jacaranda intricata	1.00	16,251	9,993	8,125	7,153	5,532	1	0.886	0.841	0.815	0.764
Leiothrix echinocephala	0.93	13,273	12,738	12,246	11,911	8,993	1	0.990	0.980	0.973	0.907
Lessingianthus eitenii	1.00	32,759	23,002	18,457	16,771	14,002	1	0.915	0.866	0.846	0.809
Lessingianthus irwinii	1.00	27,484	19,247	16,285	14,919	13,776	1	0.915	0.877	0.858	0.841
Lessingianthus souzae	1.00	35,654	29,195	28,425	27,529	27,377	1	0.951	0.945	0.937	0.936
Lessingianthus stoechas	0.82	64,825	51,900	50,569	48,893	43,890	1	0.946	0.940	0.932	0.907
Lessingianthus venosissimus	1.00	34,734	24,154	21,370	19,946	17,120	1	0.913	0.886	0.871	0.838
Lessingianthus zuccarinianus	0.95	16,133	12,438	11,132	10,134	9,214	1	0.937	0.911	0.890	0.869
Leucochloron foederale	1.00	20,311	12,918	10,642	9,877	8,409	1	0.893	0.851	0.835	0.802
Leucochloron minarum	1.00	6,262	4,990	4,535	3,750	1,592	1	0.945	0.923	0.880	0.710
Lippia bradei	0.96	14,059	11,892	11,082	9,684	5,782	1	0.959	0.942	0.911	0.801
Lippia rhodocnemis	0.85	7,178	6,798	6,589	6,404	5,699	1	0.986	0.979	0.972	0.944
Lithobium cordatum	1.00	10,535	8,744	8,419	8,131	6,637	1	0.954	0.945	0.937	0.891
Lomatozona artemisiifolia	1.00	5,452	3,899	3,149	2,565	2,433	1	0.920	0.872	0.828	0.817
Lupinus decurrens	1.00	6,112	5,851	5,563	5,282	4,738	1	0.989	0.977	0.964	0.938
Lychnophora diamantinana	0.90	14,778	14,318	13,992	13,751	9,383	1	0.992	0.986	0.982	0.893
Lychnophora gardneri	0.91	10,105	9,618	9,319	9,033	7,354	1	0.988	0.980	0.972	0.924
Lychnophora humillima	1.00	2,224	1,877	1,867	1,861	1,623	1	0.959	0.957	0.956	0.924
Lychnophora markgravii	1.00	17,065	14,848	13,729	12,935	9,150	1	0.966	0.947	0.933	0.856
Lychnophora martiana	1.00	15,553	13,584	12,613	11,301	8,103	1	0.967	0.949	0.923	0.850
Lychnophora pohlii	0.74	17,132	15,912	15,562	15,300	10,831	1	0.982	0.976	0.972	0.892
Lychnophora souzae	1.00	6,816	6,699	6,491	6,310	5,605	1	0.996	0.988	0.981	0.952
Lychnophora villosissima	0.88	12,094	11,875	11,654	11,466	8,730	1	0.995	0.991	0.987	0.922
Lychnophoriopsis candelabrum	1.00	2,464	2,263	2,151	2,126	1,308	1	0.979	0.967	0.964	0.854
Lychnophoriopsis hatschbachii	0.92	10,105	9,618	9,319	9,033	7,354	1	0.988	0.980	0.972	0.924
Malaxis jaraguae	0.74	2,865	796	781	734	641	1	0.726	0.723	0.712	0.688
Manilkara dardanoi	0.92	12,481	7,904	6,411	5,801	5,553	1	0.892	0.847	0.826	0.817
Marcetia hatschbachii	0.86	10,750	9,491	9,152	8,232	6,002	1	0.969	0.961	0.935	0.864
Miconia angelana	1.00	4,510	3,267	3,258	3,256	3,241	1	0.923	0.922	0.922	0.921
Micranthocereus auriazureus	1.00	6,850	5,994	5,782	5,171	3,911	1	0.967	0.958	0.932	0.869
Micranthocereus dolichospermaticus	1.00	4,677	2,719	2,605	1,513	1,499	1	0.873	0.864	0.754	0.752
Micranthocereus violaciflorus	0.99	5,673	5,043	4,855	4,363	3,262	1	0.971	0.962	0.936	0.871
Microlicia canastrensis	1.00	14,357	7,038	6,631	6,324	5,459	1	0.837	0.824	0.815	0.785
Microlicia flava	1.00	4,162	3,055	3,046	3,044	3,033	1	0.926	0.925	0.925	0.924
Microlicia humilis	1.00	3,748	540	458	410	390	1	0.616	0.591	0.575	0.568
Microlicia macedoi	1.00	62,894	42,379	34,986	32,925	29,513	1	0.906	0.864	0.851	0.828
Microlicia microphylla	1.00	6,816	6,699	6,491	6,310	5,605	1	0.996	0.988	0.981	0.952
Microlicia psammophila	1.00	35,654	29,195	28,425	27,529	27,377	1	0.951	0.945	0.937	0.936

Species	Proportion global range within Cerrado	Extent of Suitable Habitat (km2)					Probability of persistence				
		Original	yr 2000	yr 2010	yr 2012	yr 2014	Original	yr 2000	yr 2010	yr 2012	yr 2014
Mikania alvimii	0.96	35,408	29,090	28,000	26,957	26,754	1	0.952	0.943	0.934	0.932
Mikania glabra	0.88	10,447	10,154	10,070	10,003	6,880	1	0.993	0.991	0.989	0.901
Mikania hartbergii	1.00	2,125	1,873	1,853	1,763	1,363	1	0.969	0.966	0.954	0.895
Mikania neurocaula	0.81	12,980	12,252	12,080	11,824	9,642	1	0.986	0.982	0.977	0.928
Mikania warmingii	0.88	11,452	9,862	9,645	9,461	8,745	1	0.963	0.958	0.953	0.935
Mimosa acroconica	1.00	1,174	1,152	1,119	1,106	868	1	0.995	0.988	0.985	0.927
Mimosa adamantina	1.00	6,933	6,815	6,607	6,425	5,692	1	0.996	0.988	0.981	0.952
Mimosa heringeri	1.00	11,885	8,697	7,138	6,824	6,645	1	0.925	0.880	0.870	0.865
Mimosa lithoreas	0.90	4,431	2,817	2,642	2,427	1,521	1	0.893	0.879	0.860	0.765
Mimosa montis-carasae	0.85	6,816	6,699	6,491	6,310	5,605	1	0.996	0.988	0.981	0.952
Mimosa paucifolia	0.95	19,095	12,870	12,177	11,848	7,257	1	0.906	0.894	0.888	0.785
Mimosa suburbana	1.00	1,540	976	750	729	634	1	0.892	0.835	0.829	0.801
Mimosa uniceps	1.00	6,933	6,815	6,607	6,425	5,692	1	0.996	0.988	0.981	0.952
Minaria bifurcata	0.90	7,640	7,370	7,323	7,291	6,009	1	0.991	0.989	0.988	0.942
Minaria diamantinensis	0.90	7,640	7,370	7,323	7,291	6,009	1	0.991	0.989	0.988	0.942
Minaria grazielae	0.92	10,105	9,618	9,319	9,033	7,354	1	0.988	0.980	0.972	0.924
Minaria inconspicua	0.87	8,591	7,998	7,708	7,422	6,697	1	0.982	0.973	0.964	0.940
Minaria magisteriana	0.98	2,114	1,386	1,383	1,382	1,340	1	0.900	0.899	0.899	0.892
Minaria refractifolia	0.78	10,932	10,356	10,056	9,769	7,950	1	0.987	0.979	0.972	0.923
Minasia alpestris	0.91	11,272	11,075	10,864	10,679	8,101	1	0.996	0.991	0.987	0.921
Minasia pereirae	0.94	15,011	14,146	13,709	13,356	9,042	1	0.985	0.978	0.971	0.881
Minasia scapigera	0.91	11,974	11,716	11,430	11,232	8,423	1	0.995	0.988	0.984	0.916
Mitracarpus eritrichoides	1.00	6,524	4,281	3,532	3,037	2,920	1	0.900	0.858	0.826	0.818
Mostuea muricata	1.00	2,062	14	14	13	13	1	0.288	0.287	0.283	0.283
Nectomys squamipes	0.30	12,481	7,904	6,411	5,801	5,553	1	0.892	0.847	0.826	0.817
Oocephalus piranii	1.00	2,125	1,873	1,853	1,763	1,363	1	0.969	0.966	0.954	0.895
Orthophytum humile	1.00	7,326	6,337	6,051	5,488	3,886	1	0.964	0.953	0.930	0.853
Ossaea warmingiana	1.00	14,893	9,889	8,088	7,345	6,423	1	0.903	0.858	0.838	0.810
Oxalis diamantinae	1.00	6,816	6,699	6,491	6,310	5,605	1	0.996	0.988	0.981	0.952
Oxypetalum ekblomii	0.90	12,110	8,358	7,656	7,062	6,449	1	0.911	0.892	0.874	0.854
Paepalanthus ater	1.00	1,400	1,274	1,265	1,259	1,088	1	0.977	0.975	0.974	0.939
Paepalanthus hydra	0.80	938	938	938	938	931	1	1.000	1.000	1.000	0.998
Paliavana werdermannii	0.71	13,635	11,812	10,974	9,818	7,580	1	0.965	0.947	0.921	0.863
Pavonia grazielae	1.00	10,750	9,491	9,152	8,232	6,002	1	0.969	0.961	0.935	0.864
Pecluma hoehnii	0.80	7,502	2,771	2,552	2,552	2,324	1	0.780	0.764	0.764	0.746
Peixotoa andersonii	1.00	1,054	1,048	1,034	1,030	987	1	0.998	0.995	0.994	0.984
Peixotoa bahiana	1.00	28,098	21,504	19,892	17,468	16,491	1	0.935	0.917	0.888	0.875
Peixotoa barnebyi	0.82	11,737	11,525	11,287	11,090	8,321	1	0.995	0.990	0.986	0.918
Peixotoa psilophylla	1.00	9,994	6,466	4,836	4,470	4,374	1	0.897	0.834	0.818	0.813
Pellaea gleichenioides	0.80	9,988	9,642	9,432	9,245	7,673	1	0.991	0.986	0.981	0.936
Peperomia cordigera	0.73	7,640	7,370	7,323	7,291	6,009	1	0.991	0.989	0.988	0.942
Pfaffia argyrea	1.00	2,125	1,873	1,853	1,763	1,363	1	0.969	0.966	0.954	0.895
Phragmipedium vittatum	0.93	24,078	19,384	17,719	16,926	16,708	1	0.947	0.926	0.916	0.913
Phyllanthus gladiatus	0.78	6,816	6,699	6,491	6,310	5,605	1	0.996	0.988	0.981	0.952

Species	Proportion global range within Cerrado	Extent of Suitable Habitat (km2)					Probability of persistence				
		Original	yr 2000	yr 2010	yr 2012	yr 2014	Original	yr 2000	yr 2010	yr 2012	yr 2014
Phymatidium geiselii	0.73	1,987	241	233	232	176	1	0.590	0.585	0.585	0.545
Pilocarpus alatus	0.77	12,374	3,051	3,051	3,046	2,905	1	0.705	0.705	0.704	0.696
Pilosocereus aurisetus subsp. aurilanatus	1.00	1,761	1,620	1,557	1,548	724	1	0.979	0.970	0.968	0.801
Pilosocereus fulvilanatus	1.00	8,852	7,864	7,584	6,803	5,336	1	0.971	0.962	0.936	0.881
Piptolepis imbricata	1.00	708	689	675	670	612	1	0.993	0.988	0.986	0.964
Piptolepis leptospermoides	1.00	6,112	5,851	5,563	5,282	4,738	1	0.989	0.977	0.964	0.938
Pitcairnia bradei	1.00	7,326	6,337	6,051	5,488	3,886	1	0.964	0.953	0.930	0.853
Podocarpus barretoi	1.00	5,015	4,310	4,288	4,067	4,046	1	0.963	0.962	0.949	0.948
Podocarpus brasiliensis	1.00	14,021	11,089	9,607	9,054	8,816	1	0.943	0.910	0.896	0.890
Polygala franchetii	1.00	9,130	6,499	4,922	4,555	4,394	1	0.919	0.857	0.840	0.833
Polygala tamariscea	0.98	20,235	14,562	12,574	11,697	10,542	1	0.921	0.888	0.872	0.850
Pombalia strigoides	1.00	12,783	11,562	11,366	11,034	10,911	1	0.975	0.971	0.964	0.961
Prestonia solanifolia	0.87	17,722	10,306	9,798	8,914	6,515	1	0.873	0.862	0.842	0.779
Pseudolycopodiella benjaminiana	0.97	40,188	23,083	21,201	19,426	17,200	1	0.871	0.852	0.834	0.809
Pseudotrimezia concava	1.00	6,500	5,618	5,342	4,821	3,419	1	0.964	0.952	0.928	0.852
Pseudotrimezia synandra	1.00	8,227	8,106	7,898	7,716	6,699	1	0.996	0.990	0.984	0.950
Pseudotrimezia tenuissima	1.00	13,316	12,317	11,833	11,131	9,024	1	0.981	0.971	0.956	0.907
Pteroglossa hilariana	1.00	1,867	759	741	696	696	1	0.799	0.794	0.781	0.781
Richterago caulescens	1.00	1,977	1,643	1,632	1,626	1,430	1	0.955	0.953	0.952	0.922
Richterago elegans	0.95	8,344	8,223	8,016	7,833	6,814	1	0.996	0.990	0.984	0.951
Richterago petiolata	1.00	1,912	1,843	1,836	1,783	1,777	1	0.991	0.990	0.983	0.982
Richterago polyphylla	1.00	7,406	7,286	7,079	6,897	6,103	1	0.996	0.989	0.982	0.953
Schefflera gardneri	1.00	6,239	5,704	5,314	4,958	3,009	1	0.978	0.961	0.944	0.833
Scuticaria itirapinensis	1.00	1,255	328	320	285	270	1	0.715	0.710	0.690	0.681
Senaea coerulea	1.00	6,816	6,699	6,491	6,310	5,605	1	0.996	0.988	0.981	0.952
Senecio gertii	1.00	7,326	6,337	6,051	5,488	3,886	1	0.964	0.953	0.930	0.853
Senecio hatschbachii	0.87	8,923	8,819	8,771	8,742	6,668	1	0.997	0.996	0.995	0.930
Simaba glabra	0.88	26,589	16,320	15,463	14,758	11,360	1	0.885	0.873	0.863	0.808
Simaba salubris	0.91	11,738	2,541	1,990	1,276	937	1	0.682	0.642	0.574	0.531
Simaba warmingiana	0.89	9,485	6,374	5,992	5,721	2,383	1	0.905	0.892	0.881	0.708
Sinningia araneosa	0.84	2,194	394	393	387	375	1	0.651	0.651	0.648	0.643
Sinningia defoliata	0.97	50,416	31,495	28,963	26,445	22,352	1	0.889	0.871	0.851	0.816
Sinningia piresiana	1.00	3,444	950	938	883	868	1	0.725	0.722	0.711	0.708
Sphaerorrhiza burchellii	1.00	2,544	2,101	1,908	1,892	1,885	1	0.953	0.931	0.929	0.928
Spigelia cipoensis	1.00	1,519	1,195	1,186	1,181	1,071	1	0.942	0.940	0.939	0.916
Staelia hatschbachii	1.00	17,566	16,190	15,643	14,542	11,606	1	0.980	0.971	0.954	0.902
Stenandrium hatschbachii	1.00	8,151	6,996	6,565	5,947	4,098	1	0.963	0.947	0.924	0.842
Stenandrium stenophyllum	1.00	2,125	1,871	1,847	1,728	1,275	1	0.969	0.966	0.950	0.880
Stevia hilarii	1.00	4,162	3,055	3,046	3,044	3,033	1	0.926	0.925	0.925	0.924
Stigmaphyllon macedoanum	0.79	3,750	3,033	2,958	2,783	525	1	0.948	0.942	0.928	0.612
Strophopappus bicolor	1.00	3,705	2,905	2,709	2,619	2,442	1	0.941	0.925	0.917	0.901
Strophopappus ferrugineus	1.00	30,097	22,482	19,907	18,686	13,496	1	0.930	0.902	0.888	0.818
Svitramia integerrima	1.00	2,776	1,478	1,471	1,471	1,471	1	0.854	0.853	0.853	0.853
Svitramia minor	1.00	4,510	3,191	3,181	3,174	3,152	1	0.917	0.916	0.916	0.914

Species	Proportion global range within Cerrado	Extent of Suitable Habitat (km2) Original	yr 2000	yr 2010	yr 2012	yr 2014	Probability of persistence Original	yr 2000	yr 2010	yr 2012	yr 2014
Svitramia wurdackiana	1.00	5,204	3,196	3,186	3,176	3,166	1	0.885	0.885	0.884	0.883
Syagrus glaucescens	0.81	8,338	7,457	7,238	7,053	6,286	1	0.972	0.965	0.959	0.932
Syagrus mendanhensis	1.00	6,933	6,816	6,609	6,427	5,719	1	0.996	0.988	0.981	0.953
Symphyopappus uncinatus	0.96	8,227	8,106	7,898	7,716	6,699	1	0.996	0.990	0.984	0.950
Talisia subalbens	0.97	35,843	19,734	17,672	15,865	15,568	1	0.861	0.838	0.816	0.812
Thelypteris multigemmifera	1.00	919	341	336	316	312	1	0.780	0.778	0.766	0.763
Thryallis parviflora	1.00	15,865	11,086	8,945	8,170	7,279	1	0.914	0.867	0.847	0.823
Tibouchina bergiana	1.00	4,510	3,191	3,181	3,174	3,152	1	0.917	0.916	0.916	0.914
Trembleya hatschbachii	0.93	10,750	9,491	9,152	8,232	6,002	1	0.969	0.961	0.935	0.864
Trimezia exillima	1.00	9,283	9,139	8,908	8,721	7,243	1	0.996	0.990	0.985	0.940
Trimezia plicatifolia	1.00	4,350	4,103	3,903	3,727	2,609	1	0.985	0.973	0.962	0.880
Triraphis devia	1.00	35,654	29,195	28,425	27,529	27,377	1	0.951	0.945	0.937	0.936
Uebelmannia pectinifera	0.95	6,351	5,894	5,605	5,321	4,777	1	0.982	0.969	0.957	0.931
Vellozia armata	1.00	1,061	1,049	1,044	974	583	1	0.997	0.996	0.979	0.861
Vellozia barbata	1.00	1,405	1,395	1,385	1,384	1,165	1	0.998	0.997	0.996	0.954
Vellozia gigantea	1.00	2,806	1,788	1,775	1,769	1,608	1	0.893	0.892	0.891	0.870
Vellozia hatschbachii	1.00	9,286	8,843	8,630	8,443	7,149	1	0.988	0.982	0.976	0.937
Vellozia sessilis	1.00	12,557	11,587	11,467	11,174	11,137	1	0.980	0.978	0.971	0.970
Vellozia streptophylla	1.00	937	935	935	934	931	1	1.000	1.000	0.999	0.998
Vellozia subalata	1.00	3,611	2,604	2,570	2,536	1,984	1	0.922	0.918	0.915	0.861
Wedelia macedoi	1.00	2,439	1,623	1,492	1,353	596	1	0.903	0.884	0.863	0.703
Wunderlichia cruelsiana	0.95	35,408	29,090	28,000	26,957	26,754	1	0.952	0.943	0.934	0.932
Wunderlichia senae	0.83	9,165	9,004	8,794	8,611	7,473	1	0.996	0.990	0.985	0.950
Xyris coutensis	0.98	1,646	1,397	1,356	1,323	1,188	1	0.960	0.953	0.947	0.922
Xyris nigricans	1.00	1,519	1,195	1,186	1,181	1,071	1	0.942	0.940	0.939	0.916
Xyris platystachya	1.00	8,335	7,894	7,678	7,491	6,675	1	0.987	0.980	0.974	0.946
Xyris sincorana	0.72	6,500	5,618	5,342	4,821	3,419	1	0.964	0.952	0.928	0.852
Xyris uninervis	1.00	2,038	193	122	115	110	1	0.555	0.495	0.488	0.482
Zephyranthes candida	0.93	2,038	193	122	115	110	1	0.555	0.495	0.488	0.482

Appendix 2.

Here we provide extra analyses of the dynamic situation of soy production and trade – one that is inherent within any free-market commodity production and trade system, and particularly in agricultural frontiers. Our paper is based on 2011 trade data, to which we attribute biodiversity losses between 2000 and 2010, because a) there is a significant lag between conversion of native vegetation and soy export; and b) land conversion in the Cerrado is speculative and assumes a medium-long term future demand. Therefore, the demand that drives land conversion events (and associated biodiversity losses) is not only attributable to those who benefit in the immediate aftermath, but also those who benefit further into the future.

We present two sets of results that help us to understand how our results might be affected by using a 2011 'snapshot', versus some other year (or average over several years). First, we present a time series (to complement Figure 3 in the main text) showing trader/country relationships for traders exporting to Amsterdam Declaration countries between 2003 (the earliest year available) and 2011 (SI Appendix 2, Figure S3). We also look at an average for 2006-2010, in order to compare our 2011 snapshot with the preceding five year period (SI Appendix 2, Figure S4, top). This shows that the trader-country relations for 2011 were similar to those of 2006-2010, particularly for the bigger traders. Overall, patterns are similar between adjacent years, but generally show larger changes over longer time spans, highlighting the importance of multi-temporal and up-to-date analyses ahead of any interventions.

Whilst the relationships and patterns of trade between traders and countries might remain similar in terms of overall trade volumes (SI Appendix 2, Figures S3 & S4), it is likely that differences in geospatial sourcing patterns will also occur – i.e. trader x exports the same amount to country y, but sources from a different set of producers. This change will be reflected by a change in the relative biodiversity impact measure linked to any company (even though the overall metric of biodiversity loss between 2000-2010 is unchanged across the landscape). Therefore, we also present a comparison of the biodiversity losses associated with traders exporting to Amsterdam Declaration countries in 2011 versus the average trade over the 2006-2010 period. This analysis includes, therefore, these potential changes in geospatial sourcing pattern, which would be masked in an analysis of just overall volumes. As with the soy volume, comparing the 2006-2010 average biodiversity losses with the pattern for 2011 shows that the situation remains broadly similar in terms of the relative roles for major traders, thus supporting the idea that a 2011 snapshot of trade can usefully be applied to data on losses over a longer period. The data show that Granol, Bunge, Cargill and ADM had largest overall impact in both time periods, but also show an increase in Bunge's impact - relative to ADM for example - when we consider the 2006-2010 average. This also serves to demonstrates the framework's ability to create an indicator that will reflect changes over time

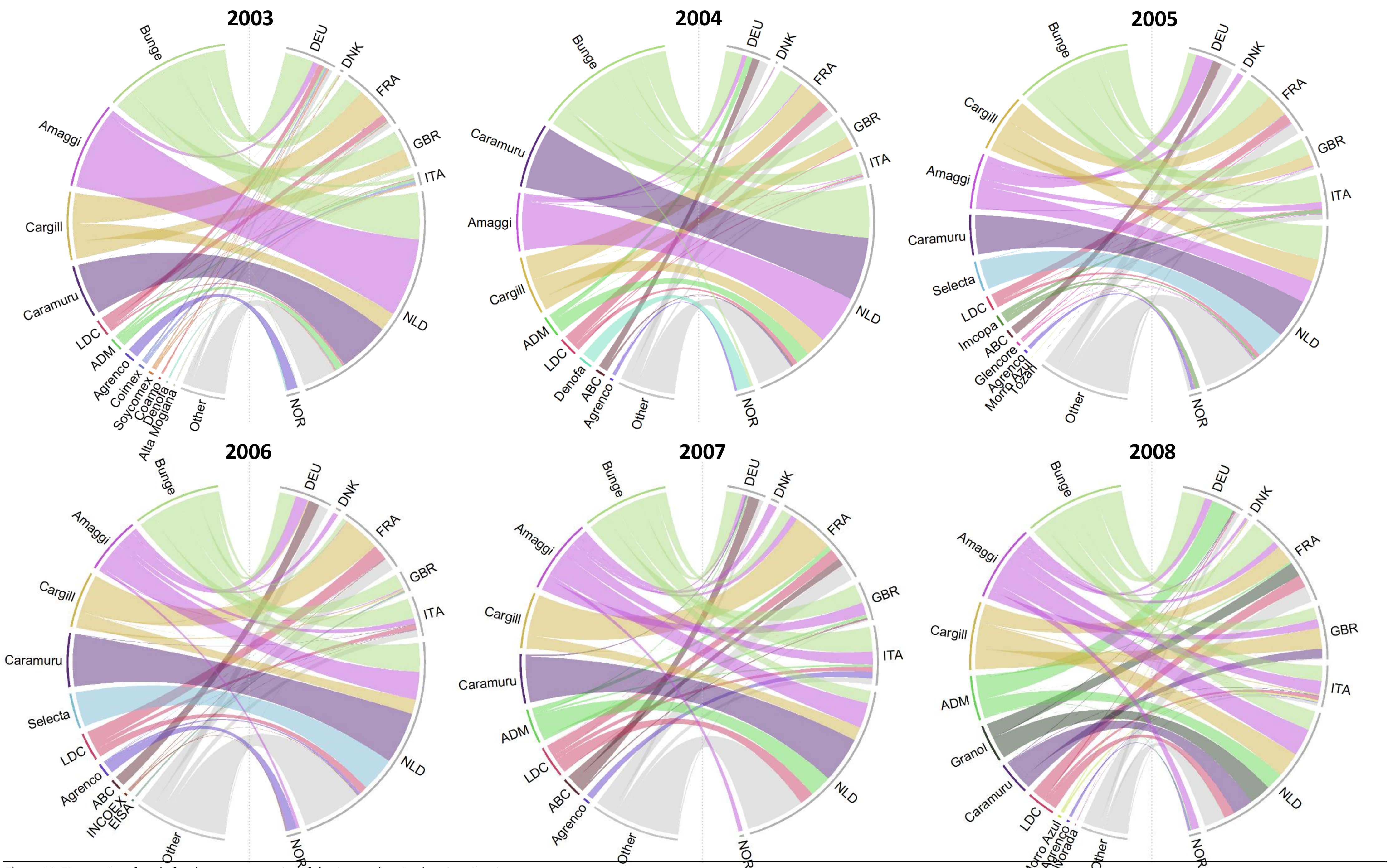

Figure S3. Time series of trade for the seven countries of the Amsterdam Declaration. Continues on next page.

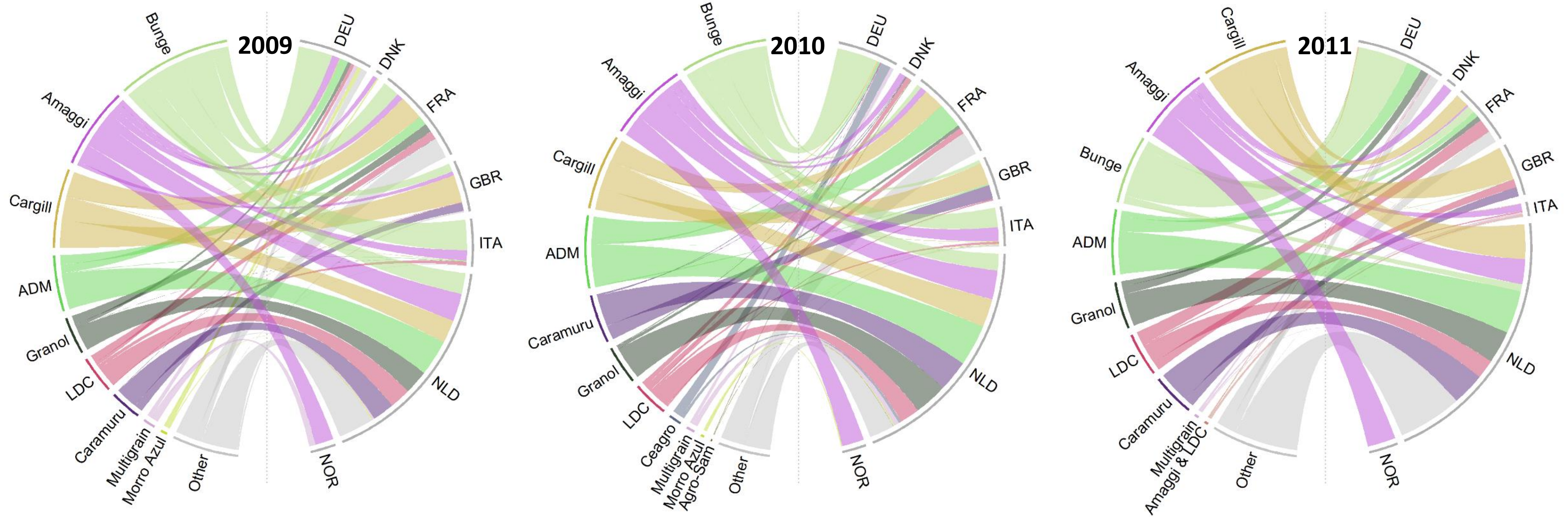

Figure S3. Time series of trade for the seven countries of the Amsterdam Declaration. The paper provides a snapshot for 2011 of a dynamic and potentially fast-changing system. Here we provide a full time series showing trader/country relationships between 2003 (the earliest year available) and 2011 (year 2011 is equivalent to Figure 3 in the main text, where traders arranged alphabetically, and zero-deforestation commitments are highlighted). For each year in the time series, traders are arranged by greatest volume (top) to least (bottom). Patterns are similar between adjacent years, but show gradual changes over longer time spans. Companies named were among the top three traders for at least one of the countries in that year; companies trading smaller volumes are aggregated and shaded grey. Country ISO 3 codes: DEU = Germany, DNK = Denmark, FRA = France, GBR = United Kingdom, ITA = Italy, NLD = Netherlands, NOR = Norway.

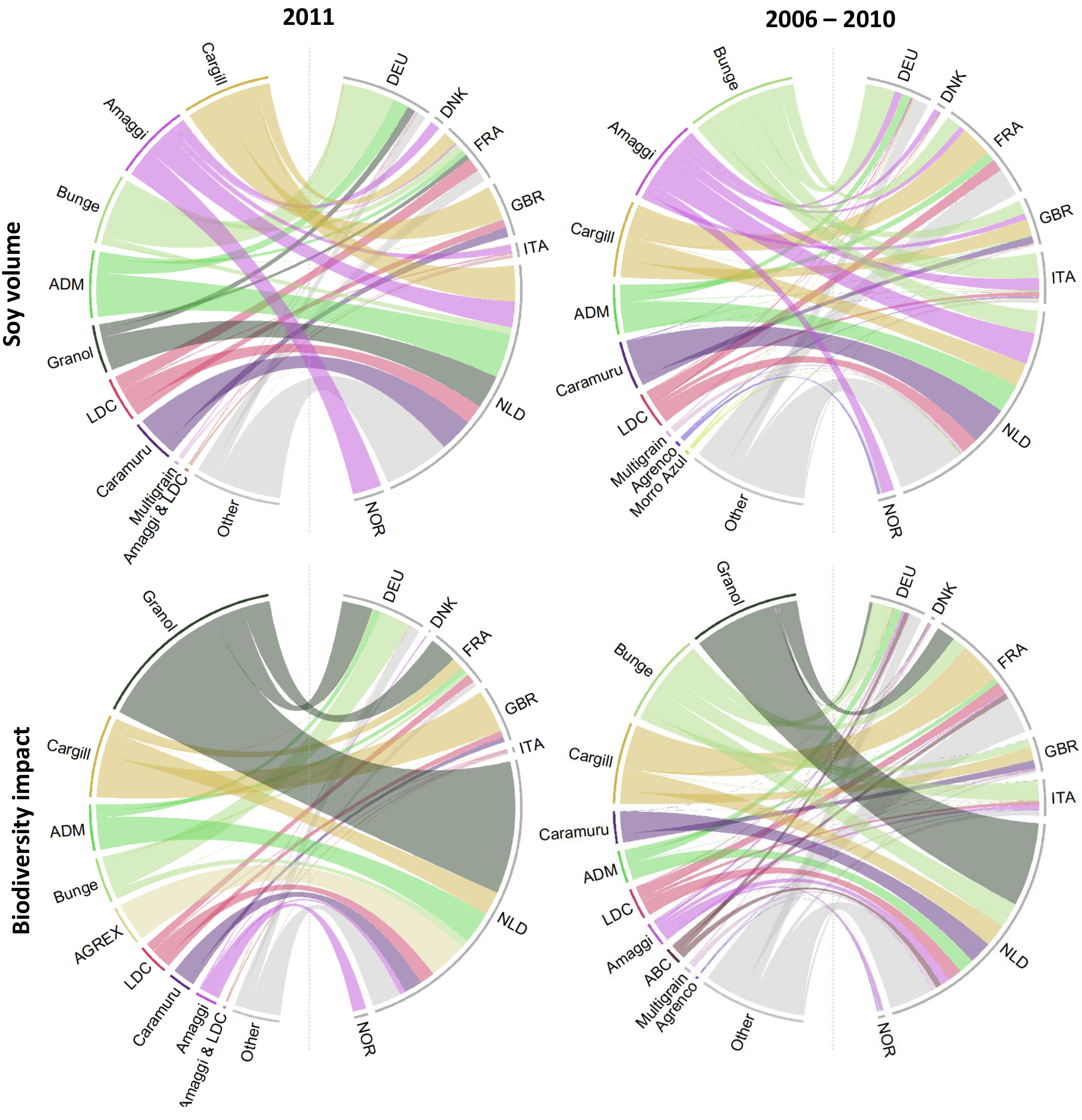

Figure S4. Comparison of 2011 snapshot (left) with the average for the preceding 5 years (2006-2010; right) for both the amount of soy traded (top) and its biodiversity impact (bottom). This shows, for our 2000-2010 study period, the greatest biodiversity losses associated with traders exporting to the Amsterdam Declaration countries. While biodiversity impacts (bottom) are considerably different to the soy volumes (top), comparing the 2006-2010 average (bottom, right) with the pattern for 2011 (bottom, left) shows that the situation remains similar. Note that for this analysis, the biodiversity loss data remain the same for each year (i.e. average losses observed from 2000-2010), and the year-to-year differences are driven by changes in the sourcing patterns of traders. Companies named were among the top three traders (of volume or of biodiversity impact risk) for the year/period for at least one of the countries; companies trading smaller volumes are aggregated and shaded grey. Country ISO 3 codes: DEU = Germany, DNK = Denmark, FRA = France, GBR = United Kingdom, ITA = Italy, NLD = Netherlands, NOR = Norway.

Appendix 3

Supplementary methods

1) Methods

Modelling approach

We compile and integrate existing data sources, linking complementary approaches to derive new information on how consumption patterns drive species declines, and shedding light on the supply chains involved (Figure S5). Existing multiregional input-output models (MRIOs) use data on inter-sectoral financial transactions to represent full global trade and consumption, but sacrifice commodity-specific detail and offer limited spatial resolution. Conversely, material flow analyses – descriptions of the physical movement of commodities – can be used to track production and trade of individual commodities, but generally capture only a portion of the supply chain (3). We therefore develop a hybridised MRIO for soy trade that combines traditional input-output analyses with the highly detailed subnational material flow data from the Spatially Explicit Information on Production to Consumption Systems (SEI-PCS) model underpinning the Trase platform (2, 4). We use these to tease out the activities of producers, traders, and consumers. We then link these models to a framework for estimating species-by-species losses of suitable habitat to derive a measure of biodiversity impact that accounts for species-specific differences in range sizes, sensitivities to land-use change and historical habitat loss (Figure S5)(5). We focus on the impacts of soy production in 2000-10 using habitat loss data for 2000-10 and soy trade data for 2011. We chose this allocation period (i.e. attributing 2000-2010 losses to 2011) because it can take several years from initial clearing of land to eventual harvesting and selling soy crop.

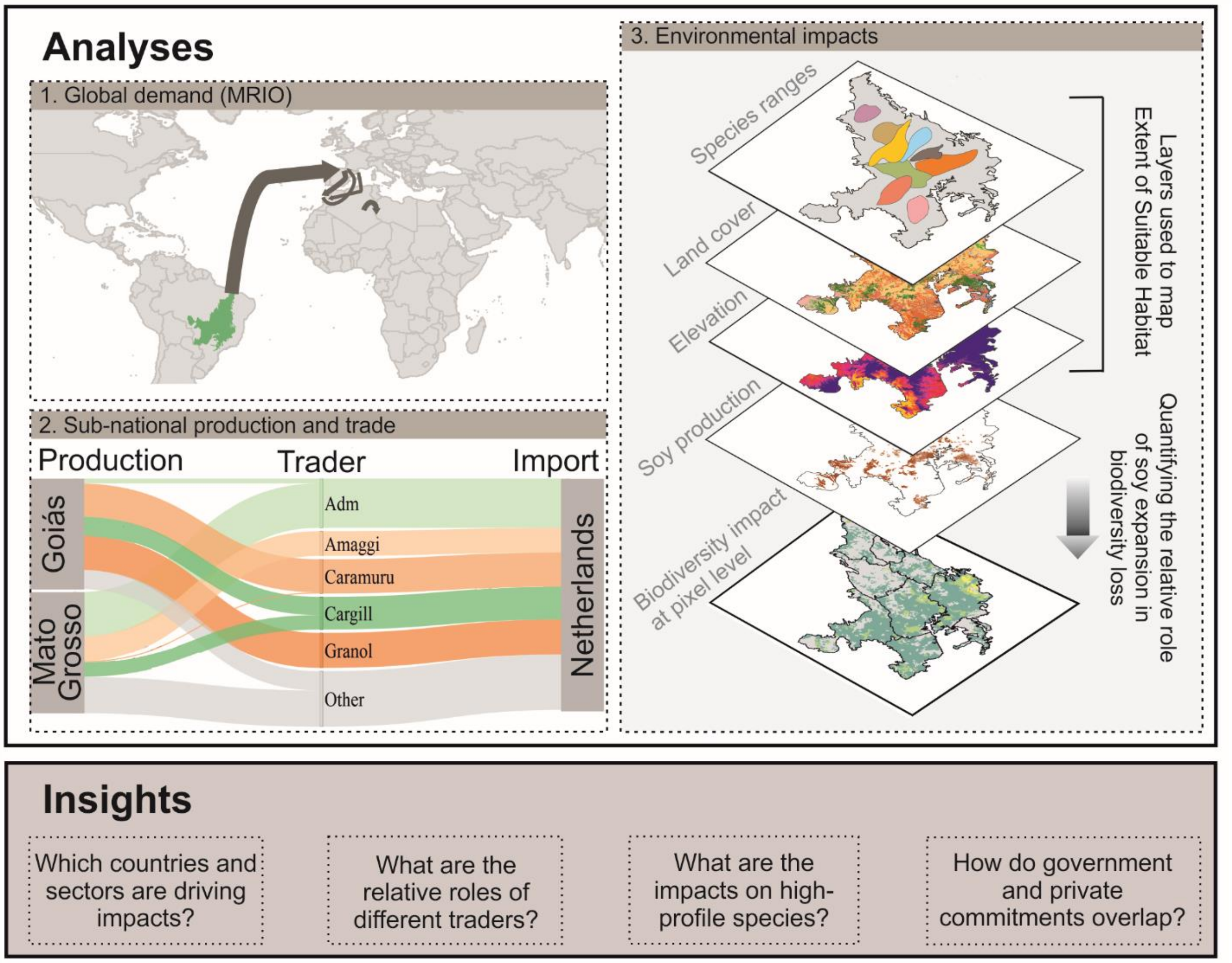

Figure S5. (Top) *Our approach uses several component models: 1. Global demand MRIO. These macroeconomic MRIO models of international trade, allow us to understand international consumption drivers (e.g. GTAP* (1)*); 2. Subnational production and trade, detailing the material flow of a particular commodity and the traders involved (e.g. the SEI-PCS model of Brazilian soy from the Trase platform* (4)*); and 3. Environmental impacts, to understand the environmental impacts of commodity production systems (e.g. on biodiversity* (5)*). Together, these models allow credible comparisons of impacts in different production locations, and provide detail on the actors and consumption patterns involved.* ***(Bottom)*** *We highlight four insights on: a) countries and sectors driving impacts; b) relative roles of the different traders involved; c) impacts on high-profile species; d) alignment of commitments between different actors.*

We use the Input-Output Trade Analysis model (IOTA), which combines commodity-level data, in physical units, with sectoral-level expenditure data, in monetary units, to represent the entire supply chain through to final consumption whilst retaining high-resolution information on origin of production (6). Within this model, we use material flow data to link subnational production to the country of import, via exports (accounting for re-exports – the reported export of a previously imported commodity –to avoid allocation of sourcing to an intermediate trade partner). Next, in conjunction with national-scale FAO data on processing of primary commodities into secondary commodities and other uses, we allocate domestic supply (production plus imports, minus exports) to derived commodities and applications (7). We then link the use of primary and derived commodities into a global MRIO using relative in-country expenditure between relevant sectors, to construct a hybridised MRIO model, IOTA, as described in Croft *et al*. (6) and here (SI Appendix 3). The global MRIO is based upon the Global Trade Analysis Project (GTAP), which details financial interdependencies between 57 sectors in 140 regions (1). We used the GTAP9 database, with 2011 as our reference year. We chose GTAP over other MRIOs for its agricultural sector categorisations, which form a good basis for agrifood applications from a production to consumption perspective, alongside

its good country/region resolution. However, for other applications we could substitute alternative MRIO models into the framework.

For our case study of Brazilian soy, production and material flow data for 2011 are from the Trase platform (2, 4). Underpinned by customs declarations and shipping records, this models the physical movement for a range of commodities linked to deforestation, from municipality of production (in the case of Brazilian soy) through regional logistic hubs (e.g. processing facilities and shipping ports) to the country of import (4). These data record both the mass being traded and also ownership details – i.e. the exporting and importing companies – which in turn allows detailed analysis of actors' roles in the supply chain (8).

For comparison of direct imports versus final consumption (Figure 1d), we calculate the biodiversity impact of direct soy imports using data from the Trase platform, which tracks commodities to the country of first import. We plot these against the outputs of the IOTA model – with biodiversity allocated in proportion to commodity flows – thereby accounting for re-exports and embedded consumption to estimate the sectors and countries of final consumption. To calculate the relative impact of different sectors (e.g. dairy, beef and other meat in Figure 1e), we sum, across all municipalities, the final consumption that is allocated to each sector. We highlight "cattle meat", "other meat" and "dairy" (GTAP sector codes: cmt, omt, and mil) because of the relative importance of the livestock sector as a driver of soy production. All other sectors are aggregated together. Methods for constructing the IOTA trade model are described in detail in Croft *et al*. (6).

These trade models provide information on the amount of commodity produced in a particular location (in this case, Brazilian municipality) and its subsequent flow through a supply chain. We then extend the framework to capture impacts on biodiversity. This requires two steps: first, the mapping of commodity production areas, and second, estimating impacts on biodiversity for those areas (5). For the former, we use maps from Gibbs *et al.* (9), who estimate soy expansion between 2003 and 2013. For the latter, we refine extent of occurrence maps for endemic and near-endemic amphibian, bird, mammal and plant species (40 amphibians, 22 birds, 22 mammals and , 324 plants with >70% range within Cerrado, hereafter referred to as 'endemics'), using information on habitat preferences and altitudinal ranges to derive extent of suitable habitat (ESH) models (for more detail, see (5) and SI Appendix 3). For plants, we clip extent of occurrence to include only natural vegetation categories, as defined by the Brazilian Institute of Geography and Statistics. For vertebrates, we use information on species-specific habitat preferences and elevation ranges from IUCN (10). We use a digital elevation model to identify, for each species, areas within both its elevation range and its extent of occurrence (11). We then estimate ESH from within this area using land cover data for 2000 and 2010 from IBGE (12), merged with maps of annual soy expansion from Gibbs *et al*. (9). See SI Appendix 1, Table S4 for a summary of species included and changes in ESH and persistence likelihood.

Biodiversity losses associated with areas of soy expansion between 2000 and 2010 were then calculated using a unit-less 'conservation score' that captures the non-linear cumulative effect of historical habitat loss on the local persistence of a species; the marginal loss of a suitable 250 m pixel is estimated by calculating the conservation score, P, for each species: $P=E^z$, where E is the remaining proportion of the original ESH, and z is analogous to an extinction coefficient, which was set at $z=0.25$ for this study, but can be varied (5, 13). To estimate original ESH for vertebrates prior to large-scale cultivation, we use a map of 'original' vegetation cover for the Cerrado (ca. 16th century) (14, 15), and in the absence of more detailed information for plants, we use the entire geographic range to delineate the original extent. For each pixel, we sum the conservation score for all species whose ESH overlapped it in 2000. In addition, we map the ESH of migratory birds, separately for their resident, breeding and non-breeding ranges, allowing us to incorporate differences in their seasonal

requirements into estimates of biodiversity impact (5). The framework can be used to map impacts on individual species, or any combination of species. We also provide an alternative set of results in SI Appendix 4, Figs. S8 and S9 using just the extent of suitable habitat to estimate impacts (effectively z=1). Our results remain qualitatively very similar and do not change our conclusions.

Finally, we calculate biodiversity losses between 2000 and 2010 and aggregate to municipality level. We then use these estimates to rescale the modelled raw soy equivalents that enter the trade model at the municipality of production. Impact is then attributed to supply chain actors (as intermediaries) and to final consumers in proportion to the volume of raw soy equivalents traded or consumed.

Geographic analyses are conducted in ArcGIS at 250 m resolution and all other analyses in R (16, 17), with code available in Dataset S1. Chord diagrams are constructed using the circlize package in R (18).

2) Trase SEI-PCS model

We summarise the supply chain mapping approach used by Trase to connect subnational regions of production to trading countries and countries of import around the world. The description below is drawn from the Trase manual and description of data sources (19, 20). Please refer there for full details and refer to the website, trase.earth (2), to download freely-available data.

The Spatially Explicit Information on Production to Consumption Systems (SEI-PCS) model is a form of enhanced material flow analysis that was first described by Godar *et al.* (4) in 2015. It has evolved beyond its initial scope and ambition, including through the integration of additional fine-scale data sets on production, taxation, logistics, sanitary inspections and trade data.

Three capabilities of the Trase approach set it apart from other approaches to supply chain mapping:

1. It systematically links individual supply chain actors to specific, subnational production regions, and the sustainability risks associated with those regions;
2. It identifies the individual companies that export, ship and import a given traded commodity; and
3. It covers all of the exports of a given commodity from a given country of production.

The starting point for applying the SEI-PCS approach to a specific country and commodity is national-level export data, linking countries of production to downstream traders and countries of import. This higher-level analysis explores material flows and associated sustainability impacts, risks and performance measures at national level, as well as providing an entry point for more detailed work on poorly studied geographies and sectors. The SEI-PCS approach is then applied to map subnational trade flows, discriminating production regions down to the lowest level of government administrative unit that the data and the complexity of the supply chain allow. Often this is defined by the availability of production data at subnational scales.

For the Brazilian soy SEI-PCS model, the basic structure of the supply chain is defined by highly disaggregated customs declarations compiled from official sources. Trase maps exports of soy beans, cake, and oil (but not of animal feed or biodiesel, given that these involve other raw products that can be used in varying proportions, and in which soy could be substituted for example by corn or animal fat, respectively. Instead, this is dealt with when we employ the hybridised MRIO (IOTA) in the next step.

Data sources

Trase makes use of per-shipment customs and shipping data for sustainability research. Central to the SEI-PCS approach is the use of multiple independent data sets to “triangulate” flows of traded commodities from regions of production via trading companies to countries of import. SEI-PCS typically uses data collected for other purposes, such as customs records and maritime shipping contracts, tax registration data, logistic ownership and capacity, sanitary and commodity movement controls, and production data. A common feature that allows these diverse data sets to be linked is the identification of a specific amount of a commodity in a specific location and time, as well as its ownership. The SEI-PCS approach is highly flexible and can be adapted to include new data sets that can help add further detail and/or provide additional validation for individual commodity flows.

No private or confidential information has been used to construct the Brazilian soy SEI-PCS model. All data sources are either publicly available (including from government and industry websites and repositories) or available for purchase (for example from trade intelligence companies). All data are from official sources, whether government agencies or industry representatives, and obtained directly from these sources or via private vendors that have been granted access to it. The trade data sources, such as per-shipment customs declarations or bills of lading, is integrated with other data sources in a final SEI-PCS data model, and the detailed shipment-level information is aggregated per year, per commodity equivalent, per country of destination and per jurisdiction of sourcing, which makes it impossible to reverse engineer the raw data or to get similar per shipment detail as is found in the originals. A description of all the data sets currently used in Trase, and their sources, is in Table S5, below.

Table S5. *Data sources used to construct and validate the Brazilian soy SEI-PCS model. Table adapted from Trase 2019* (20).

**Agricultural data**		
Soy production	Municipal soy production (tonnes)	Brazilian Bureau of Statistics (IBGE) (https://sidra.ibge.gov.br)
Soy yield	Average municipal soy yield (tonnes/ha) from annual municipal production survey	Brazilian Bureau of Statistics (IBGE) (https://sidra.ibge.gov.br)
Agricultural land used for soy	Percentage of agricultural land in the municipality that is soy	Brazilian Bureau of Statistics (IBGE) (https://sidra.ibge.gov.br)
**Trade data**		
Customs declarations	Tax documents recording shipments compiled by the Brazilian government	Various sources
Bills of lading	Maritime contracts between exporters and importers guaranteeing volume, value and destination of shipments	Various sources
Crushing facility data	Soy crushing and processing facilities	ABIOVE (http://www.abiove.org.br/)
Silos	Silo records	CONAB SICARM (http://www.conab.gov.br/detalhe.php?a=107 8&t=2)
CNPJs	Brazilian Tax Identification numbers linking companies with municipalities and authorized commercial activities.	Various sources

3) IOTA model

The Input-output Trade Analysis (IOTA) model is a hybridized physical-financial multi-regional input-output (MRIO) model (6). It utilises commodity specific data to track the production and trade of commodities and their direct derivatives, a re-export algorithm to account for the trade of goods by intermediate countries, domestic use data to determine the sectors of the economy utilising a country’s domestic supply (production + imports - exports) of these commodities, and finally an MRIO to model the remainder of supply chain through to final consumption. IOTA currently employs the GTAP MRIO which has 140 global regions and 57 economic sectors, though any other MRIO could be used, subject to a redistribution of physical flows to the appropriate monetary sectors which differ across MRIO datasets. The GTAP MRIO is constructed as described by Peters *et al.* (21) and is

particularly appropriate in this case for the sectoral detail that it offers for agricultural commodities. An overview of the model is given in Figure S6 and a more detailed flowchart describing how different data sources are used within the model is given in Figure S7.

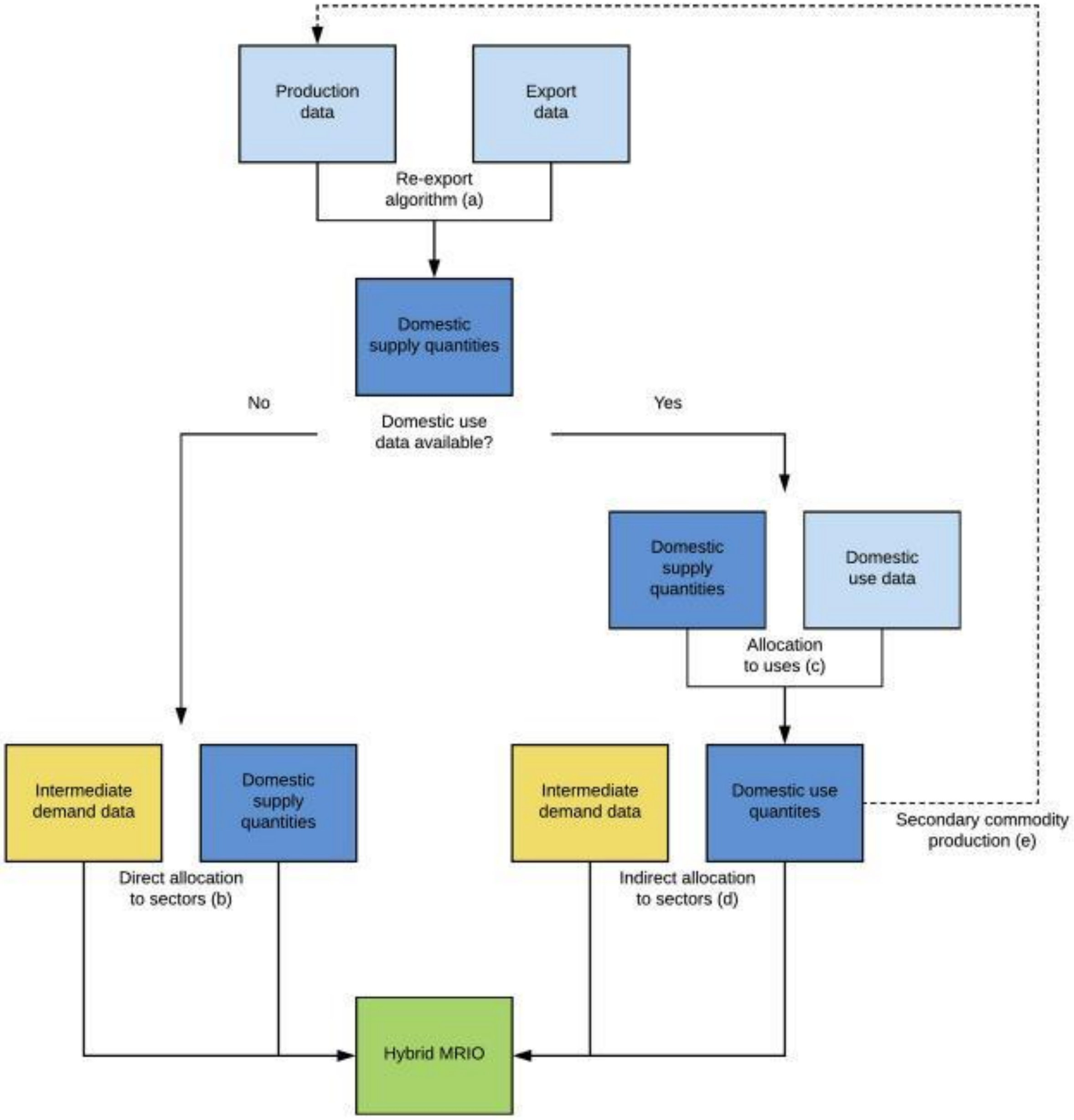

Figure S6. *Simplified visualisation of model framework and data structure from Croft et al. (6). Light blue denotes input data in physical units; dark blue calculated data in physical units; yellow input data in monetary units; green modelled outputs. Production and trade data are used to obtain country-level domestic supply quantities via the re-export algorithm (a). Where use data are not available, relative expenditure within the MRIO (intermediate demand) is used to distribute domestic supply directly to economic sectors (b). Where use data are available, supply is distributed to use categories (c). Domestic use quantities are used in conjunction with the relative expenditure within the MRIO to more accurately distribute domestic supply to appropriate economic sectors (d). Where processing to secondary commodities does occur, this is fed back in as production of a "new" commodity at the start of the model framework (e). Full description of steps (a)–(e) provided in Croft et al. (6).*

FAO national level production and bilateral trade data (22) for soybean, soybean oil and soybean cake are combined with the subnational level data for Brazil (with Brazil at the national level effectively being replaced with a number of subnational jurisdictions).

For raw soybean, this is fed through IOTA's re-export algorithm (see re-export algorithm in section 3, below) to link points of production to points of final import. This algorithm retains the total quantity of soy within the system, while prohibiting countries and subnational jurisdictions that produce no soy to export. In the case of intermediary trading countries, trade from there to other countries is allocated back to the original source of the imports.

Domestic use data from FAO's commodity balance sheets (7) provides the country level quantity of unprocessed soybean which is processed into oil and cake, as well as quantities used for food, feed, seed and "other" uses. The latter volumes of unprocessed soybean are connected to the MRIO in the appropriate sectors (e.g. use for feed is allocated to sectors associated with the rearing of animals) within importing countries according to relative expenditure by these sectors on the appropriate sector associated with soy production.

The processed soy (oil and cake) is removed from the system, and these are run individually through the re-export algorithm (see section 3, below), with the countries where the soy is processed acting as points of production. This can then be linked back to the origin of the raw soy, to link the final imports of oil and cake products to the point of production of the raw soy from which they were derived. The commodity balances sheets again provide information on the use of the oil and cake, and this is used to allocate them within the appropriate sectors within the importing countries according to relative expenditure by the different sectors on the appropriate parent sector.

At this point three hybridised MRIOs have been produced (one for raw soy, oil and cake), with information pertaining to the origin of the raw commodity (including subnational resolution for Brazil). Conventional MRIO methods are then employed to calculate the Leontief inverse (23), from which final demand can be used to calculate the embedded consumption of soy, oil and cake across all economic sectors and countries of purchase. The results for soy, oil and cake are then combined to give total embedded soy consumption, retaining the links to point of origin.

We focus on the impacts of soy production in 2000-10 using habitat loss data for 2000-10 and soy trade data for 2011. We chose this allocation period (i.e. attributing 2000-2010 losses to 2011) because it can take several years from initial clearing of land to eventual harvesting and selling soy crop. In the Cerrado, it is common that people acquire and prepare land with the goal of then selling or leasing it. Acquisition, preparation and selling the land is viable within this 10 year period. We could, alternatively, have used an 'average trade' across the 2000 to 2010 period (or any other year/period from 2003-2017 for which we have data). All data are freely available for download.

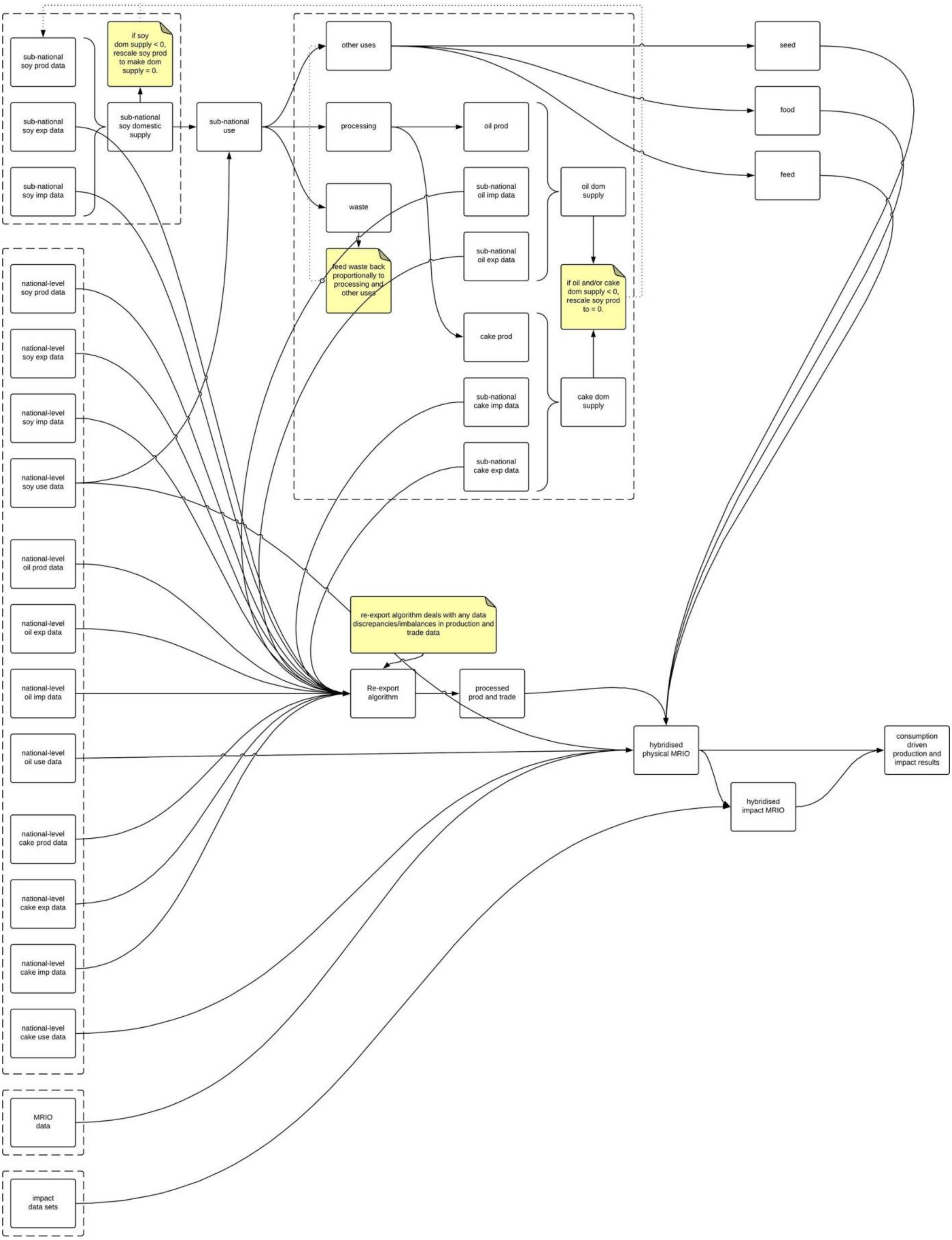

Figure S7. *Visual representation and flow diagram of all data sources and intermediate steps in the application of IOTA to subnational Brazilian soy production from Croft et al. (6). The final outputs are hybridised versions of the GTAP MRIO, linking consumption from economic sectors across different countries and regions to localised soy production and impacts.*

4) Re-export algorithm

The following description is adapted from Croft *et al.* (6).

For a given commodity, country level production can be considered as a vector, P, listing production quantity (e.g. by mass) for n countries, such that

$$P = \begin{bmatrix} p_1 \\ p_2 \\ \cdots \\ p_n \end{bmatrix} \quad \text{(Eqn. S1)}$$

Similarly, exports can be considered as a matrix, E, where e_{ij} denotes the export from country i to country j, such that

$$E = \begin{bmatrix} 0 & e_{12} & \cdots & e_{1n} \\ e_{21} & 0 & \dots & e_{2n} \\ \vdots & \vdots & \ddots & \vdots \\ e_{n1} & e_{n2} & \dots & 0 \end{bmatrix} \quad \text{(Eqn. S2)}$$

The diagonal entries are all zero valued since a country does not "export" to itself; the quantity of domestically produced goods that remain within the producing country will instead be derived from production and export totals.

Combining production and trade data allows for domestic supply, D, to be calculated with information of origin. Domestic supply is simply defined as production plus imports minus exports. Here d_{ij} is the quantity of country j's domestic supply which originates from country i.

$$D = \begin{bmatrix} d_{11} & d_{12} & \cdots & d_{1n} \\ d_{21} & d_{22} & \dots & d_{2n} \\ \vdots & \vdots & \ddots & \vdots \\ d_{n1} & d_{n2} & \dots & d_{nn} \end{bmatrix} \quad \text{(Eqn. S3)}$$

where d_{ii} is domestic supply of domestically produced goods, that is

$$d_{ii} = p_i - \textstyle\sum_{j \neq i} d_{ij} \quad \text{(Eqn. S4)}$$

If D is constructed directly from available production and trade data, the issue of re-exports manifests itself in the form of erroneous allocation of traded commodities to the wrong country of origin. Whilst in some instances such allocations can be effectively invisible (i.e. there is no obvious indication that an incorrect allocation of production has occurred), in others it creates a clear issue to overcome as the calculated domestic supply of domestically produced goods, d_{ii}, can be negative.

Using reported production, P, and trade, E, data, the algorithm calculates domestic supply, D. The algorithm works by repeatedly performing a two-step operation for N iterations (here N=10,000). Initially D is a zero matrix, and the two-step operation is as follows:

- Step 1: 1/N of each country's annual domestic production is added to their domestic supply (i.e. $d_{ii}=d_{ii}+p(i)/N$).
- Step 2: 1/N of annual exports are moved from each country's domestic supply to the appropriate recipients. These exports are proportionally comprised of domestically produced and imported goods according to current domestic supply, and are capped by available domestic supply.

The first time Step 2 is performed, domestic supply is purely domestically produced as no trade has yet been modelled. But the domestic supply soon becomes comprised of both domestically produced and imported goods, and subsequent calculations of trade account for exports and re-exports, whilst never allowing a country to trade more than it possesses. The outcomes of this are that:

- Total global domestic supply is constrained and equal to total global production (i.e. total quantity of goods is conserved).
- No country exports more than it produces, or re-exports more than it imports (i.e. no negative domestic supply values).
- Commodity origin is linked back to the source of production, not intermediate traders (i.e. trade links true origin to final destination).

As such, rows in *D* will be zero-valued for non-producing countries, and the domestic supply for each country (columns) will only contain non-zero entries for countries where production has occurred and with who trade (direct and/or indirect) has taken place. MatLab code for the re-export algorithm is supplied (open access) as supplementary material with Croft *et al.* (6).

5) Biodiversity impact extension

We used the results of Durán *et al.* (5) and here reproduce their description of the methods. Duran *et al.* (5) is currently under review, but is also archived and freely available in the online repository bioarxiv. Whilst we think that this method improves upon the traditional linear approach of weighting species impacts only by Extent of Suitable Habitat (ESH) loss (in which losing 9 ha of habitat is considered the same for a species that has 10 ha of habitat and for a species that has 1,000 ha), we also provide analyses based on the traditional linear approach using raw ESH loss values in SI Appendix 4, Figs. S8 and S9.

Method and rational of the biodiversity footprint indicator

The approach involves three steps: i) Mapping the extent of suitable habitat for each species of interest, thus including species' distributions; ii) Estimating the marginal value of suitable habitat for each species, by estimating the reduction in their population persistence from the proportional loss of ESH due to land-cover conversion. Within this same step, combining the estimates across species to assess biodiversity impact, thereby considering quantity and variability of species; and iii) Mapping ESH within the Cerrado biome to estimate impacts of soy expansion.

i) Mapping the Extent of Suitable Habitat (ESH)

We included all endemic or near endemic species whose geographic ranges intersect the Cerrado, and for which habitat information is available (see SI Appendix 1, Table S4). We considered species with more than 70% of their global range falling within the Cerrado to be near endemics, and refer to both throughout the text as 'endemic' unless specifically noted. We also included analyses for two iconic and charismatic species – the Giant Anteater (*Myrmecophaga tridactyla*) and the Maned Wolf (*Chrysocyon brachyurus*). Every grid cell in a species' range in the region can be coded as suitable if: (i) the cell is within the geographic range of the species, and (ii) the local environment is within the species' known habitat preferences (in terms of land cover, elevation). The latter requires the harmonisation, in consultation with experts, of the categories of the available land-cover map with those used to describe species' habitat preferences. Coding of the suitability of cells was repeated in the same way for various points in time using environmental data appropriate for each time. Each time period is assessed against a benchmark time – in this case 'pre-industrial' landcover. For migratory species, ESH was mapped separately for each species' resident, breeding and non-breeding ranges, based on seasonal differences in their habitat preferences. This accounts for seasonal variation

in species' habitat requirements and where populations rely disproportionately on a small area to breed (for example), this is then accorded greater weight.

ii) Estimating the marginal value of suitable habitat

The next step involves calculating, for each species, the remaining proportion of its initial benchmark ESH within the study area at each subsequent point in time. Changes in ESH are then used to derive a non-linear persistence score, *P*, which captures the cumulative effect of habitat loss on the likelihood of the species' persistence in the study region:

$$P = E^z \qquad \text{(Eqn. S5)}$$

where *E* is the remaining proportion of the original ESH, and *z* is the extinction coefficient. This is analogous (at the level of a single species; Thomas *et al.* (24)) to the community-level species-area curve ($S=cA^z$). We propose its use here based on the conjecture that the conversion of given absolute area of suitable habitat after a species has lost a small amount of its initial benchmark ESH is likely to reduce the probability of the species' persistence less than if the same area of suitable habitat was lost after much of the initial ESH had already been converted. As an increasing number of studies have demonstrated, historical habitat loss has important cumulative and delayed effects on biodiversity (25), and ignoring such effects by assuming, for example, a linear relationship between habitat loss and species' persistence (equivalent to a z=1 in Eqn. S5), can result in severe underestimation of the impacts of habitat loss on biodiversity.

In addition, range size (here estimated by ESH), is a key factor in extinction risk and is also closely correlated with population size (26, 27). Therefore, reduction in species distribution is expected to affect populations' persistence (28). Here we used proportion of ESH (rather than absolute area), which allows us to standardize impacts across species. The initial benchmark ESH reflects the historical distribution size, when we do not expect land cover changes to have had an impact on probability of persistence (i.e. probability of persistence = 1; see below for more details). Consequently, probability of persistence for species with restricted ranges is expected to decline faster with habitat loss since the loss of one unit of absolute area means a higher proportional loss than for a widespread species. It is worth noting, however, that further work is required to establish empirically how the absolute and proportional area losses of individual species are related to probability of persistence. As yet, there is no standard method for such a calculation.

Once *P* has been estimated for two or more time points, the effect of any habitat loss on each species' likelihood of persistence within the study area can be calculated as *ΔP*, the corresponding difference in P-values:

$$\Delta P = [(E_{t0})^z - (E_{t1})^z] \qquad \text{(Eqn. S6)}$$

where E_{t0} and E_{t1} are the remaining proportions of ESH at *t0* and *t1*, respectively.

For migratory species, an overall ΔP_{mig} score is calculated from ΔP_{mig} scores derived separately for the species' breeding and non-breeding ESH. In order to estimate the total change in a migratory species' persistence score, a multiplicative effect can be assumed, as previously suggested by empirical and theoretical studies (29, 30):

$$\Delta P_{mig} = P_{b,t0} \times P_{nb,t0} - P_{b,t1} \times P_{nb,t1} \qquad \text{(Eqn. S7)}$$

where P_b and P_{nb} are the persistence scores within the breeding and non-breeding ranges, respectively. This approach accounts for an interactive effect between populations' likelihood of persistence along the migratory movements - an important effect to consider in biodiversity impact

quantifications (for further discussion of the implications of this approach see Appendix S1 and Figure S1.1 in Supporting Information for Durán *et al.* (5)).

To estimate global-level impacts when the study region itself is not global, each species' ΔP-values are weighted by the proportion of its global geographic range falling within the study region. The weighted ΔP of each species (including migratory ones) is then assigned to individual cells to derive the marginal value of the loss of suitable habitat, MV, for each cell converted over a given time interval. Thus, for a period of time *t0* to *t1*, the marginal value of the loss of suitable habitat within cell *j* (belonging to a set of converted cells *R*), for the weighted ΔP score of species *k*, $MV_{t0\text{-}t1, j, k,}$ can be represented as:

$$MV_{to_{t1},j,k} = \Delta P_k \frac{w_k}{R_k} \qquad \text{(Eqn. S8)}$$

where *R* is the total number of cells converted from suitable to unsuitable for that species in the period *t0* to *t1*, and *w* is the weight of species *k* (representing, for example, the proportion of its geographic range falling within the study region). The resulting distribution maps of marginal loss values for individual species are then overlaid and values summed across species to obtain, for each cell, an aggregated biodiversity impact metric. Using maps of administrative boundaries (e.g. municipalities), we aggregate the cell-level impact values to give totals for administrative units of interest. The resulting aggregated impact value will therefore reflect the level of richness, historical habitat loss and endemicity of those species occurring within the administrative unit.

[Please also refer to SI Appendix 4 for supplementary analyses using a linear relationship between ESH and biodiversity impact (i.e. z=1)]

iii) Mapping ESH within the Cerrado biome to estimate impacts of soy expansion

We applied the approach outlined above to the specific case of the expansion of soy cultivation in the Cerrado over the period 2000 – 2010. After selecting amphibians, birds, mammals and plants whose current ranges intersected the Cerrado boundary (31) and for which habitat information was available, we produced habitat suitability models to obtain 40 ESHs for amphibians, 22 for birds and 22 for mammal species, each at 250 m x 250 m resolution (the resolution of the best available land cover maps for Brazil with which land-use change can be quantified consistently; (12)). Based on information on habitat associations and elevation limits obtained from the IUCN Habitats Classification Scheme, we refined the historical geographic range (Extant, Probably Extant, Possibly Extinct, Extinct and Presence Uncertain) of each vertebrate species (10, 32) using a digital elevation model and land cover maps (11, 12, 31). The 14 categories of the land-cover map were harmonised with the 74 habitat preference levels (for more details see Appendix S2 and Table S2.1 in Supporting Information for Durán *et al.* (5)).

We also produced habitat suitability model maps for 324 endemic plant species whose ranges intersect the Cerrado. In the absence of more detailed information on species' habitat requirements, we refined their geographic ranges (33) using information on vegetation types. We assumed that only those vegetation categories classified as 'natural' by the Brazilian Institute of Geography and Statistics (IBGE) were suitable for these species, while other semi-and non-natural categories were unsuitable.

We applied our approach to IBGE land cover maps for the years 2000 and 2010. For vertebrates, we used a map of original vegetation cover for the Cerrado as our initial benchmark (c.a. 16th century; (14)), to estimate ESH prior to large-scale cultivation for each species. For plants, the original ESH was delineated by the geographic range intersecting the study region. Finally, to quantify the biodiversity impact of soybean expansion, we used cumulative soy expansion from Gibbs *et al.* (9) to map direct

expansion of soy into natural vegetation (where soy production occurred within three years of natural vegetation conversion).

Appendix 4

Supplementary analyses: results from alternative biodiversity method

Our metric uses a power law (see methods above) to weight losses of habitat according to their original extent and losses to date. The sensitivity of analyses into soy impacts on biodiversity was tested by Durán *et al.* (5) (see supplementary information therein) and the logic is further set out in Balmford *et al.* (13):

> "*Changes in population viability depend not just on the number of individuals added or removed, but also on the initial population size (losing 1,000 individuals will clearly reduce the viability of a starting population of 1,010 far more than that of a population of 10,000, for example). The increasing impact on viability of losing one individual increases as population size decreases, implying that the relationship between population size and viability is concave. We therefore follow others (Phalan, 2009; Thomas et al., 2004) in converting changes in suitable habitat area or population size into changes in population viability using a power-law function with an exponent less than one. Adoption of a similar approach in the context of the IUCN Red List criteria (Clements, Bradshaw, Brook, & Laurance, 2011) was criticised (e.g., Akçakaya et al., 2011) for not using a meaningful baseline to define the point at which a population is certain to persist (i.e., for which viability = 1), and above which viability cannot increase with population size. We tackle this here by expressing the population sizes under our agricultural scenarios relative to those under our pre-agricultural baseline. We then translate these into viabilities, averaged across all K species of a taxon in a region: where mean species population viability under different land-use scenarios is calculated by raising each species' population size relative to the pre-agricultural population size to the power z, and taking the mean of this quantity across all species.*"

However, we also report here (Figs. S8, S9 below, and also included in Appendix 1, Tables S1 and S4) our results using raw ESH losses, where impact is directly proportional to loss of ESH (effectively z=1). This might be a more appropriate metric in some circumstances - for instance in estimating numbers of individuals (rather than how that population size translates into a probability of extinction).

Results

The results shown in Figures S8e and S8f (linear) are marginal in terms of difference from Figures 1e and 1f (power law) in the main text. Germany's relative impact increases by 0.2% for example, while others stay close to the calculations using the power law method. Likewise, the difference between Figure S9 (linear) and Figure 4 (power law) in the main text is marginal. The distribution between countries is similar and for a single species is identical. The biggest difference is that the 'severity' of impacts appears larger for the species when assessed as a proportion of ESH loss, whereas we estimate lower associated impacts on species' probability of persistence.

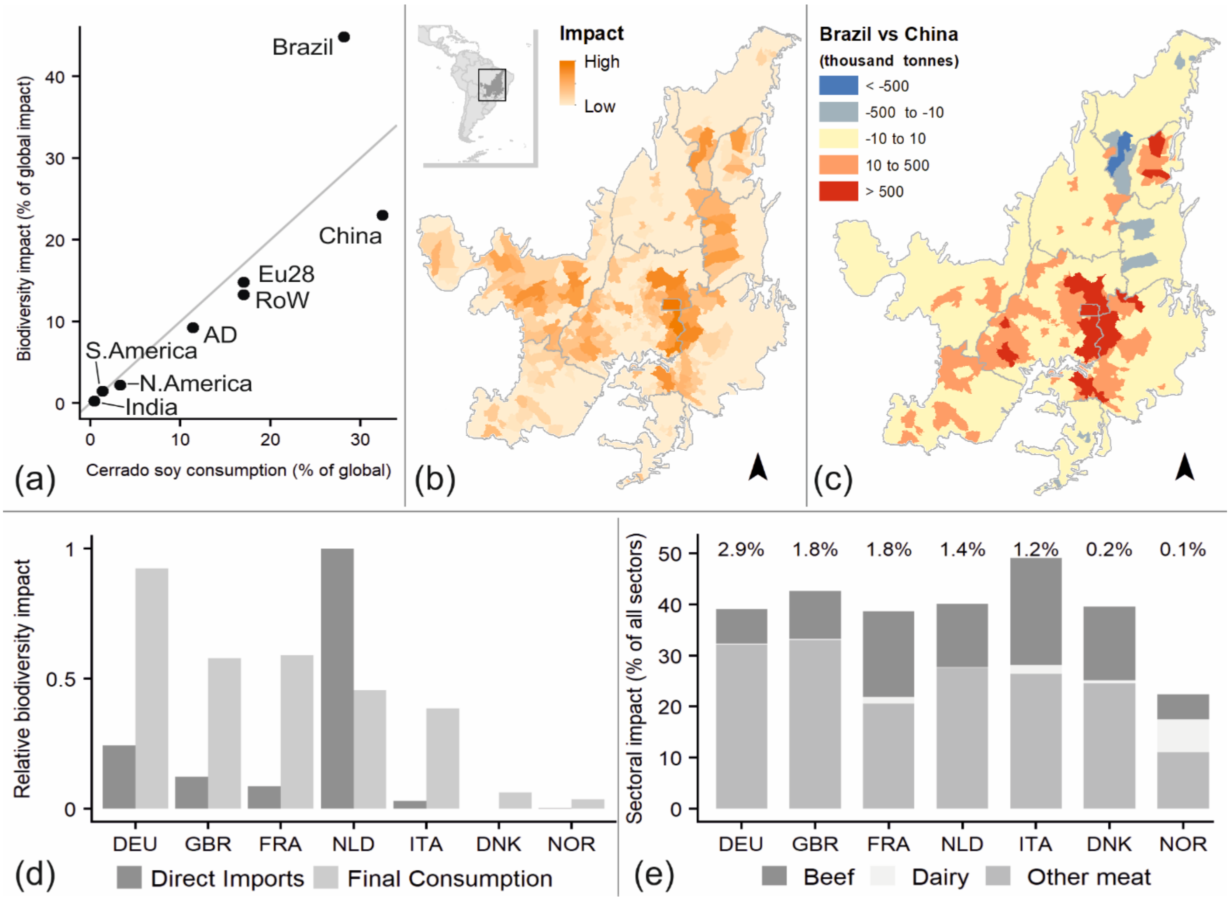

Figure S8. (alternative to Figure 1 in main text). *For b and c, figures are identical. For a, d and e, biodiversity impact is measured simply as the loss of extent of suitable habitat.* ***(a)*** *Impact of soy grown in the Cerrado on endemic biodiversity (as a percentage of global impacts of soy in the Cerrado), plotted against embedded consumption of Cerrado-sourced soy (as a percentage of global Cerrado-sourced soy consumption) for the seven countries of the Amsterdam Declaration (AD), Brazil, the 28 countries of the European Union (EU28), China (including Hong Kong and Taiwan), India, North America, South America, and the Rest of the World (RoW). Grey line indicates mean global impact per unit of soy consumption.* ***(b)*** *Spatial pattern of our index of endemic biodiversity loss within the Cerrado during the period 2000-2010;* ***(c)*** *The difference (tonnes) between production for domestic consumption (all Brazil) and Chinese consumption. Negative values (blue) are municipalities where production for Chinese consumption exceeds production for Brazilian consumption. Positive values (orange/red) are municipalities where production for Brazilian consumption exceeds production for Chinese consumption;* ***(d)*** *Comparison of the relative biodiversity impact attributed to soy that is directly imported to AD countries and the impact that is attributed to final consumption within those countries (i.e. the latter accounts for both re-exports and embedded consumption);* ***(e)*** *Sectoral and country-wise differences for AD countries showing the relative impact of three key soy-linked sectors as a percentage of each country's consumption of soy across all sectors combined. Value above bar indicates the relative importance of each country to global biodiversity impacts of Cerrado-sourced soy.*

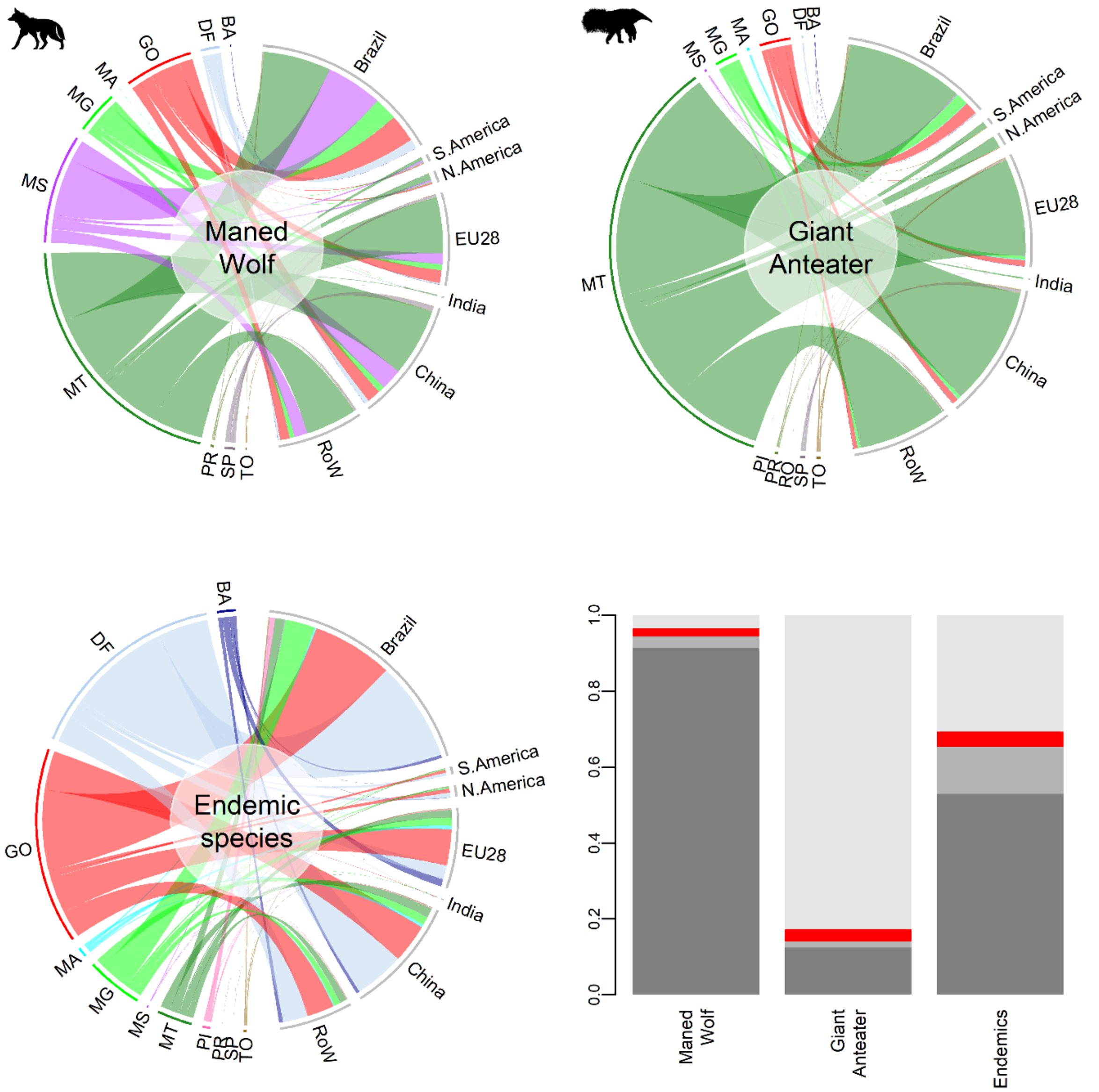

***Figure S9.* (alternative to Figure 3 in main text).** *Chord diagrams showing impacts on extent of suitable habitat due to soy expansion between 2000 and 2010 for two charismatic species (top) and summed for all endemics (bottom left). Losses are calculated for each municipality according to the total embedded flows of soy and then aggregated to state-level for visualisation. Chords show the flow from states on the left hand side (BA=Bahia, dark blue; DF=Distrito Federal, grey; GO=Goiás, red; MA=Maranhão, cyan; MG=Minas Gerais, light green; MS=Mato Grosso do Sul, purple; MT=Mato Grosso, dark green; PI=Piauí, pink; PR=Paraná, dark olive green; RO=Rondônia, brown; SP=São Paulo, dark grey; TO=Tocantins, gold) through to the country or region of final consumption on the right hand side (Brazil; South America; North America, European Union; India; China; Rest of World). The proportion of remaining suitable habitat within the Cerrado for the two species (bottom right) and the mean for all endemic and near endemic species. Light grey: suitable habitat lost from pre-industrial era to year 2000; red: losses during the 2000 to 2010 study period (as represented in the chord diagrams); medium grey: losses between 2010 and 2014; dark grey: remaining suitable habitat in 2014* (5).

References for appendices

1. GTAP Data Bases: GTAP 9 Data Base Available at: https://www.gtap.agecon.purdue.edu/databases/v9/default.asp [Accessed February 13, 2018].

2. Trase (2018) Transparency for Sustainable Economies (Trase) 2018 SEI-PCS Brazil soy (v.2.3). Available at: trase.earth [Accessed June 20, 2019].

3. Bruckner M, Fischer G, Tramberend S, Giljum S (2015) Measuring telecouplings in the global land system: a review and comparative evaluation of land footprint accounting methods. *Ecol Econ* 114:11–21.

4. Godar J, Persson UM, Tizado EJ, Meyfroidt P (2015) Towards more accurate and policy relevant footprint analyses: tracing fine-scale socio-environmental impacts of production to consumption. *Ecol Econ* 112:25–35.

5. Durán AP, et al. (2018) Putting species back on the map: devising a practical method for quantifying the biodiversity impacts of land conversion. *www.biorxiv.org*. Available at: Available as pre-print archive: https://www.biorxiv.org/content/early/2018/10/18/447466 [Accessed January 18, 2019].

6. Croft SA, West CD, Green JMH (2018) Capturing the heterogeneity of sub-national production in global trade flows. *J Clean Prod*. doi:10.1016/j.jclepro.2018.08.267.

7. FAOSTAT FAOSTAT commodity balance sheets. Available at: http://www.fao.org/faostat/en/#data/BC [Accessed August 28, 2018].

8. Trase Yearbook 2018 *Trase Yearb 2018*. Available at: https://yearbook2018.trase.earth/ [Accessed August 29, 2018].

9. Gibbs HK, et al. (2015) Brazil's soy moratorium. *Science* 347(6220):377–378.

10. IUCN (2017) *IUCN Red List of Threatened Species. Version 2017-1*.

11. United States Geological Survey (2006) United States Geological Survey (USGS). 2006 Shuttle Radar Topography Mission 3 arc second version 2.0. Publisher: Global Land Cover Facility, University of Maryland, College Park, Maryland. Available at: http://www.landcover.org/data/srtm [Accessed November 15, 2016].

12. Instituto Brasileiro de Geografia e Estatística (IBGE) Cobertura e uso da terra do Brasil 2000, 2010, 2012, 2014. Available at: ftp://geoftp.ibge.gov.br/informacoes_ambientais/cobertura_e_uso_da_terra/mudancas/vetores/ [Accessed August 28, 2016].

13. Balmford B, Green RE, Onial M, Phalan B, Balmford A (2019) How imperfect can land sparing be before land sharing is more favourable for wild species? *J Appl Ecol* 56(1):73–84.

14. Instituto Brasileiro de Geografia e Estatística (IBGE) (2004) Mapa de Vegetação do Brasil.

15. Buurman M, et al. Description of the GLOBIOM-BRAZIL database available in the REDD-PAC WFS server. Available at: http://www.redd-pac.org/reports/wfs_globiom_brazil_1April2015.pdf [Accessed August 30, 2018].

16. R Core Team (2018) *R: A language and environment for statistical computing* (R Foundation for Statistical Computing, Vienna, Austria) Available at: https://www.r-project.org.

17. ESRI (2011) *ArcGIS Desktop: Release 10.* (Environmental Systems Research Institute, Redlands, CA).

18. Gu Z, Gu L, Eils R, Schlesner M, Brors B (2014) circlize implements and enhances circular visualization in R. *Bioinformatics* 30(19):2811–2812.

19. Trase (2018) *Supply chain mapping in Trase: Summary of data and methods* (Transparency for Sustainable Economies) Available at: http://resources.trase.earth/documents/Trase_supply_chain_mapping_manual.pdf [Accessed June 20, 2019].

20. Trase (2019) *Trase data sources: SEI-PCS Brazil soy (v.2.3), SEI-PCS Paraguay soy (v.1.1), and Paraguay Beef (v.1.0)* (Transparency for Sustainable Economies) Available at: http://resources.trase.earth/documents/Trase-data-sources_release_may_2019.pdf [Accessed June 20, 2019].

21. Peters GP, Andrew R, Lennox J (2011) Constructing an environmentally-extended multi-regional input–output table using the GTAP database. *Econ Syst Res* 23(2):131–152.

22. The Food and Agriculture Organization FAOSTAT data. Available at: http://www.fao.org/faostat/en/#data [Accessed June 20, 2019].

23. Miller RE, Blair PD (1985) Input-Output Analysis: Foundations and Extensions (Englewood Cli\t hs, NJ, Prentice-Hall).

24. Thomas CD, et al. (2004) Extinction risk from climate change. *Nature* 427(6970):145.

25. Krauss J, et al. (2010) Habitat fragmentation causes immediate and time-delayed biodiversity loss at different trophic levels. *Ecol Lett* 13(5):597–605.

26. Harris G, Pimm SL (2008) Range size and extinction risk in forest birds. *Conserv Biol* 22(1):163–171.

27. Blackburn TM, Gaston KJ, Quinn RM, Arnold H, Gregory RD (1997) Of mice and wrens: the relation between abundance and geographic range size in British mammals and birds. *Philos Trans R Soc Lond B Biol Sci* 352(1352):419–427.

28. Nature IU for C of, Commission ISS, Nature IU for C of, Commission NRSS (2001) *IUCN Red List categories and criteria* (IUCN).

29. Lockwood JA (2009) *Locust: the devastating rise and mysterious disappearance of the insect that shaped the American frontier* (Basic Books).

30. Iwamura T, et al. (2013) Migratory connectivity magnifies the consequences of habitat loss from sea-level rise for shorebird populations. *Proc R Soc B Biol Sci* 280(1761):20130325.

31. Instituto Brasileiro de Geografia e Estatística (IBGE) Brazilian Territorial Area. Available at: https://ww2.ibge.gov.br/english/geociencias/cartografia/default_territ_area.shtm [Accessed February 8, 2018].

32. BirdLife International and NatureServe (2016) *Bird species distribution maps of the world.* (BirdLife International, Cambridge, UK and NatureServe, Arlington, USA).

33. Martinelli G, Moraes MA (2013) Livro vermelho da flora do Brasil.
